# Robustly forecasting maize yields in Tanzania based on climatic predictors

**DOI:** 10.1038/s41598-020-76315-8

**Published:** 2020-11-12

**Authors:** Rahel Laudien, Bernhard Schauberger, David Makowski, Christoph Gornott

**Affiliations:** 1 Potsdam Institute for Climate Impact Research (PIK), P.O. Box 60 12 03, 14412 Potsdam, Germany; 2 National Research Institute for Agriculture, Food and Environment (INRAE), UMR 518 AgroParisTech Université Paris-Saclay, 16 rue Claude Bernard, 75231 Paris Cedex 05, France; 3 Agroecosystem Analysis and Modelling, Faculty of Organic Agricultural Sciences, University of Kassel, Mönchebergstraße 19, 34109 Kassel, Germany

**Keywords:** Climate sciences, Climate change, Projection and prediction, Plant sciences

## Abstract

Seasonal yield forecasts are important to support agricultural development programs and can contribute to improved food security in developing countries. Despite their importance, no operational forecasting system on sub-national level is yet in place in Tanzania. We develop a statistical maize yield forecast based on regional yield statistics in Tanzania and climatic predictors, covering the period 2009–2019. We forecast both yield anomalies and absolute yields at the sub-national scale about 6 weeks before the harvest. The forecasted yield anomalies (absolute yields) have a median Nash–Sutcliffe efficiency coefficient of 0.72 (0.79) in the out-of-sample cross validation, which corresponds to a median root mean squared error of 0.13 t/ha for absolute yields. In addition, we perform an out-of-sample variable selection and produce completely independent yield forecasts for the harvest year 2019. Our study is potentially applicable to other countries with short time series of yield data and inaccessible or low quality weather data due to the usage of only global climate data and a strict and transparent assessment of the forecasting skill.

## Introduction

To support food security planning in face of unfavourable weather conditions, accurate yield forecasts at sub-seasonal to seasonal timescales, i.e. from weeks to months ahead, are important. Such yield forecasts can be used for early warning so that actions can be taken before the disaster occurred. On a regional and national scale, a seasonal yield forecast allows to adjust food imports so that food shortages in case of harvest losses or failures can be alleviated. If available on a high spatial resolution, a yield forecast can also inform farm management practices, such as fertilizer use, and decisions on sale prices and grain storage^1,2^.

Despite the importance of yield forecasts, as expressed by the Division of National Food Security of the Ministry of Agriculture in Tanzania (personal communications), no operational yield forecasting system on sub-national level exists up to now. The Famine Early Warning Systems Network (FEWS-net), which provides regular food insecurity reports for East Africa, stopped providing yield forecasts for Tanzania since December 2017. Ogutu et al. (2018)^3^ proposed a yield forecasting system for East Africa but this system relies on model simulations that were not validated with observational data at a subnational scale so that the accuracy of the forecast is unknown. Liu and Basso (2020)^2^ provide a yield forecast for three case studies in Tanzania based on a process-based model with a lead time of 14–77 days. They calibrate and validate the forecast using survey data at field scale. However, the practical application of this forecast for an operational forecasting system is hampered due to the limited spatial (three regions) and temporal scale (year 2017) and the high potential implementation costs (necessity to collect survey data for every new forecast).

In this study, we provide a statistical yield forecast for the whole country covering the time period from 2009 to 2019, which can be operationalized in a technical and cost-efficient way. We provide a within-season forecast of maize yields at the subnational level about 6 weeks before harvest and relate the yields to weather and sea surface temperature data. We focus on maize (*Zea mays *L.), as this is the main staple crop in Tanzania^4^. We conduct a strict model validation—not only comprising of an out-of-sample validation, but also an out-of-sample variable selection—to assess the forecasting skill.

## Results

### The influence of weather on maize yield variability

**Figure 1:**
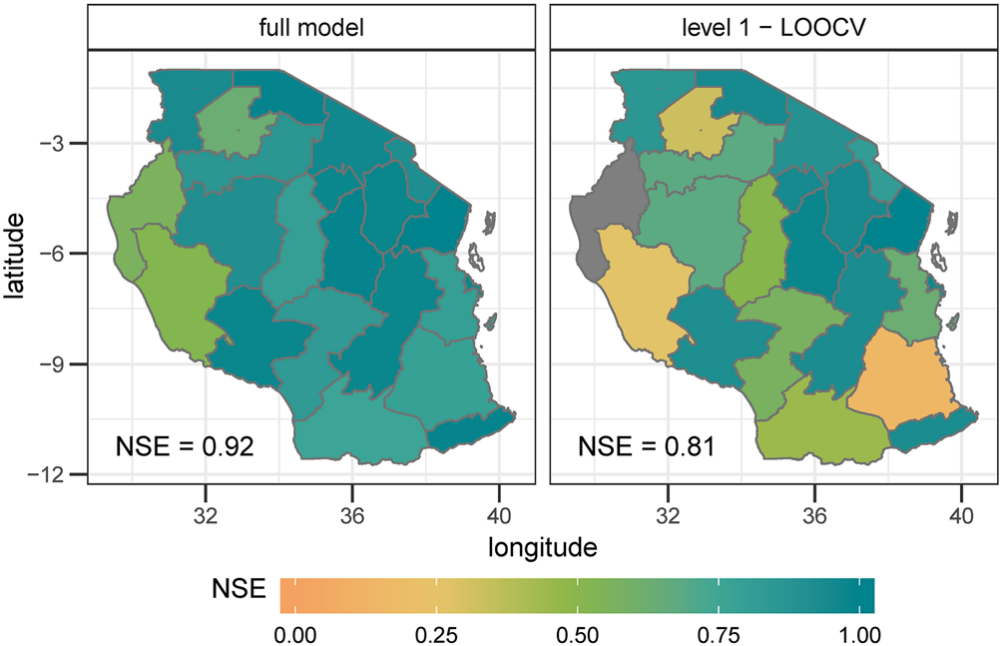
Model performance for yield anomalies from 2009 to 2018 based on selected region-specific weather variables measured in the Nash–Sutcliffe efficiency coefficient (NSE). The NSE can range from 1 (100% agreement between observed and modelled data) to − ∞; a value of zero can be interpreted in a way that nothing of the observed variability can be explained by the model. The left panel shows the model performance when the complete time series for each region is included. The right hand panel shows the performance for the leave-one-out cross validation. The colour grey indicates an NSE lower than 0. The median NSE over all regions of Tanzania is shown in the left corner of the panels.

**Figure 2:**
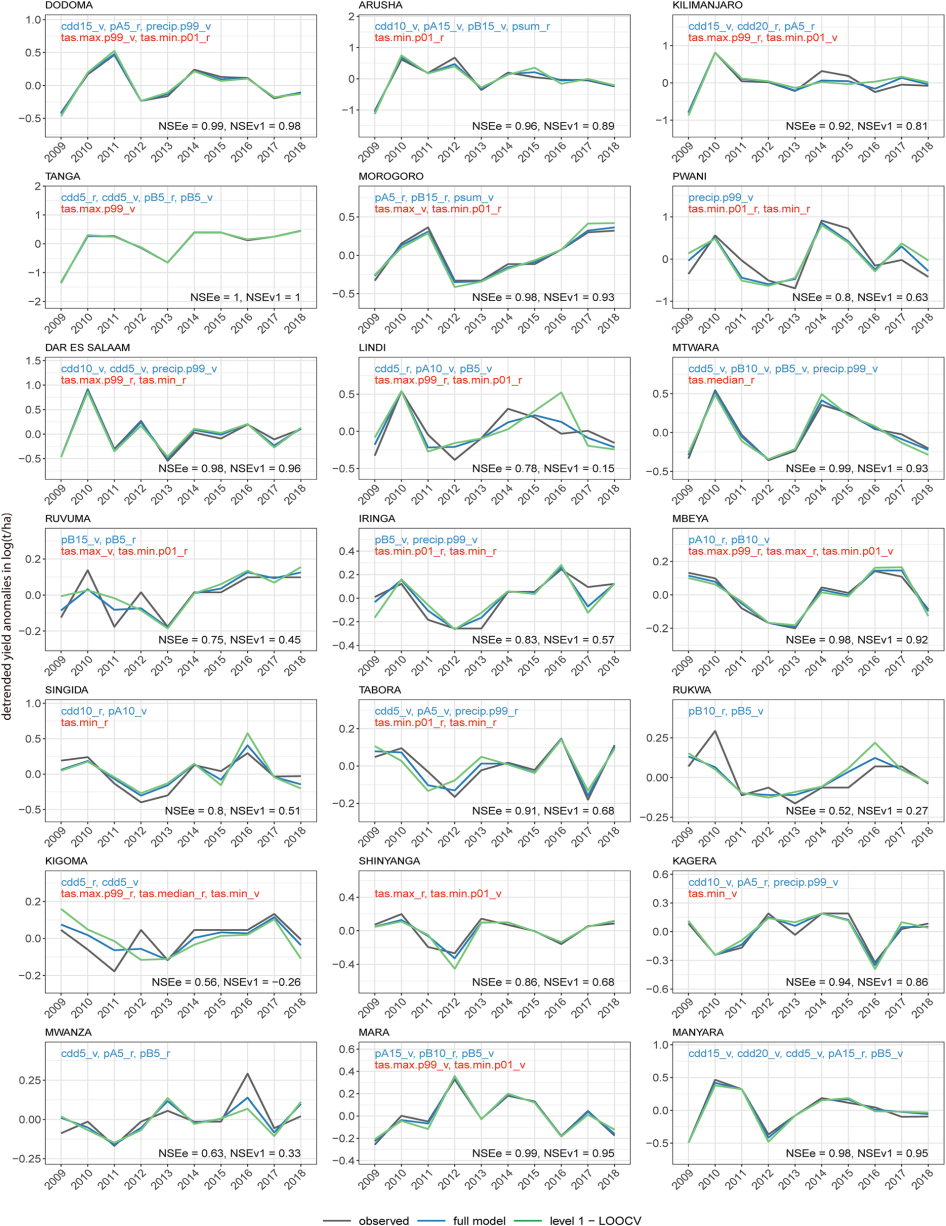
Observed and modelled maize yield anomalies in Tanzanian regions based on region-specific selected weather inputs. Grey lines show observed yield anomalies, blue lines show modelled anomalies using the full time series for model development, green lines indicate out-of-sample (level 1-LOOCV) estimates. The y-axis shows detrended yield anomalies in logarithmic form. The names of the inputs are shown in blue for precipitation and red for temperature-related variables. Explanations of abbreviations for input names are provided in supplemental information (SI) Tables 1, 2 and 3.

In the first step, we assessed how weather influenced maize variability from 2009 to 2018. The median Nash–Sutcliffe efficiency coefficient^5^ (NSE) of all models for the estimation results is 0.92 (coined ‘NSEe’ hereinafter). The NSE of the level 1 validation (coined ‘NSEv1’) is 0.81. The models show a high performance for almost the whole country (18 out of 21 regions have an NSEv1 of higher than 0.3; Fig. 1). Extreme years, like e.g. the high yields in 2010 in Dar es Salaam and Manyara or low yields in 2016 in Kagera, as well as average yields can be reproduced by the models (Fig. 2).

**Figure 3:**
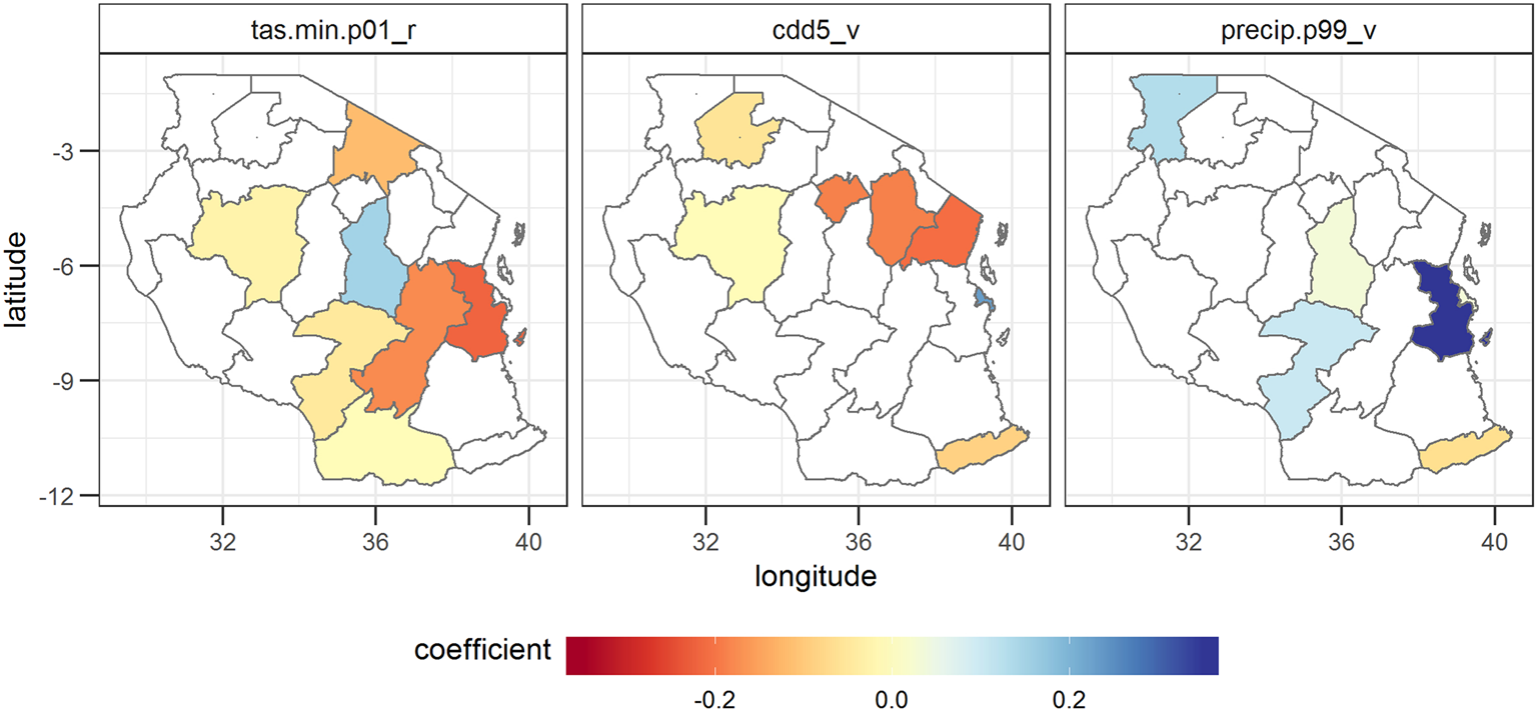
Estimated regression coefficients for the three most often selected variables. The three most often selected variables are temperature events below the 1% minimum temperature percentile in the reproductive phase (tas.min.p01_r), consecutive dry days of more than 5 days in the vegetative phase (cdd5_v) and precipitation events above the 99th precipitation percentile in the vegetative phase (precip.p99_v). Note that this analysis excludes the model coefficients with a performance lower than an NSE of 0.3 in the level 1 validation, because we assumed these models as not robust enough for further analysis. Negative (positive) values indicate that yields tend to decrease (increase) as a function of the values of the variables. The coefficients show standardized values, i.e. they show the change in yield per standard deviation of the input variable. Regions, in which the variable was not selected, are shown in white. The statistical significance of the estimated regression coefficients is shown in SI Fig. 1.

The variable selection reveals the strong influence of extreme weather events on maize yields in Tanzania. In general, maize yields seem to benefit from higher minimum temperatures, more precipitation and the absence of more than 5 consecutive dry days (Fig. 3).

In the vegetative phase, high rainfall events (precip.p99_v, i.e. precipitation events with more rain than the 99% percentile) are related with higher yields, whereas the occurrence of consecutive dry days of more than 5 days (cdd5) are mostly negatively correlated with yield. Consecutive dry days play an important role in explaining maize variability. In total, cdd5, cdd10 and cdd15 have been selected 17 times. They are mostly negatively correlated with yields, in particular in the vegetative phase. Temperature events below the 1% percentile of the minimum temperature in the reproductive phase (tas.min.p01_r) are related with lower yields—indicating that too low minimum temperatures are detrimental for maize yields.

### The influence of sea surface temperatures on maize yield variability

The usage of sea surface temperature (SST) indicators in a separate, alternative model formulation led to a median model performance of an NSE of 0.29 (level 1-LOOCV), which shows that SST variables can explain a substantial part of yield variability, but that the influence of weather is stronger.

**Figure 4:**
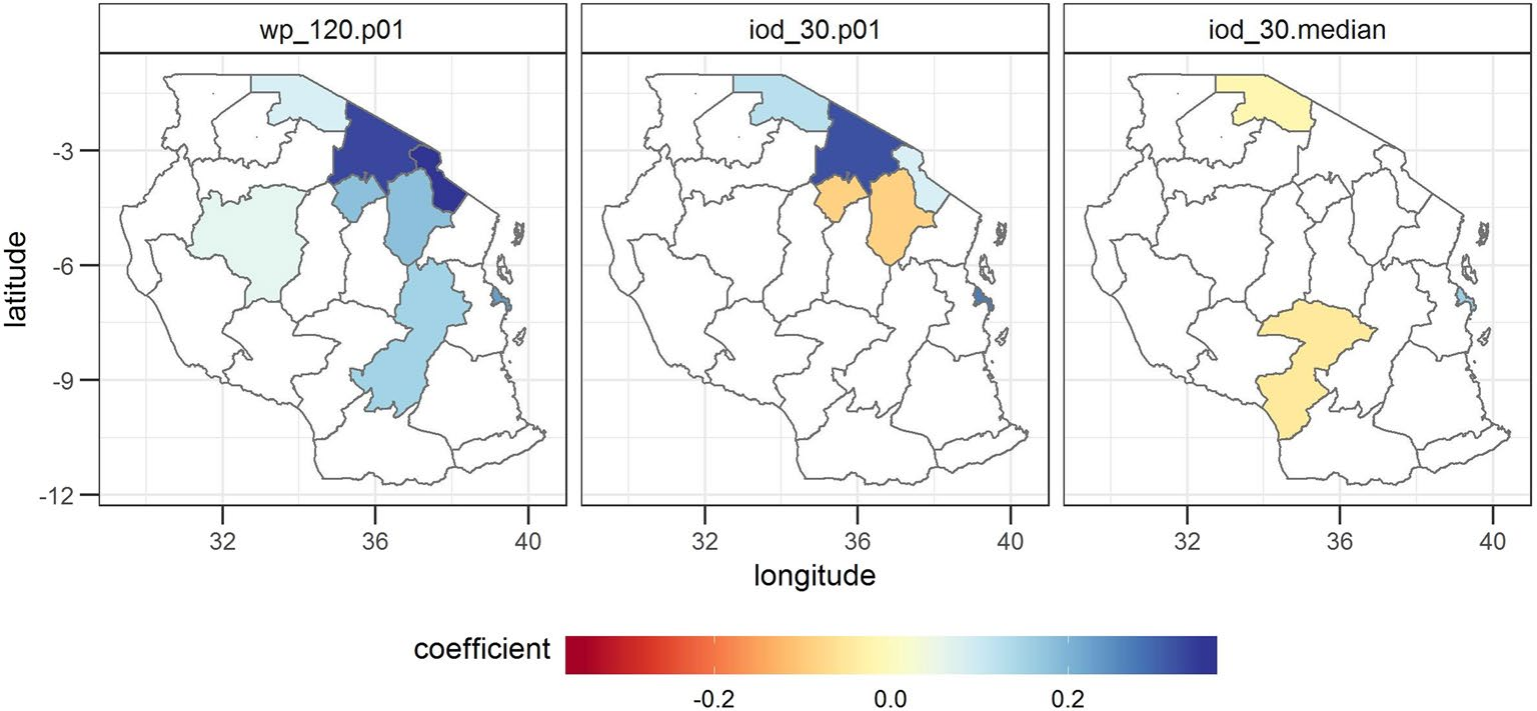
Coefficients of the most often selected sea surface temperature (SST) variables. The three most often selected variables are the number of times the SST falls below the 1% percentile of the West Pacific considering a lead time of 120 days (wp_120.p01), the number of times the SST falls below the 1% percentile of the Indian Ocean Dipole considering a lead time of 30 days (iod_30.p01) and the median SST of the IOD considering a lead time of 30 days (iod_30.median). Further explanations can be found under Fig. 3. The statistical significance of the estimated regression coefficients is shown in SI Fig. 2.

The SST of the West Pacific (WP) with a lead time of 120 days has the strongest influence on maize variability from 2009 to 2018. Maize variability shows a positive correlation with the number of times the SST falls below the 1% percentile of the WP SST (Fig. 4). The SST of the Indian Ocean Dipole (IOD) with a lead time of 30 days influences mostly the bimodal rainfall regions in Tanzania. The number of times the SST of the IOD falls below the 1% percentile is mostly positively correlated with yield, whereas the median IOD shows a mostly negative correlation with yield.

### Forecasting yield anomalies

We performed a within-season forecast by including the weather and SST variables related to the vegetative phase of the growing season. This allows us to provide a yield forecast around 6 weeks before the calculated harvest date. To assess the hindcast-based operational performance of the forecast, we include the level 1 (i.e. the out-of-sample validation) and level 2 validation (i.e. the out-of-sample variable selection validation).

**Figure 5:**
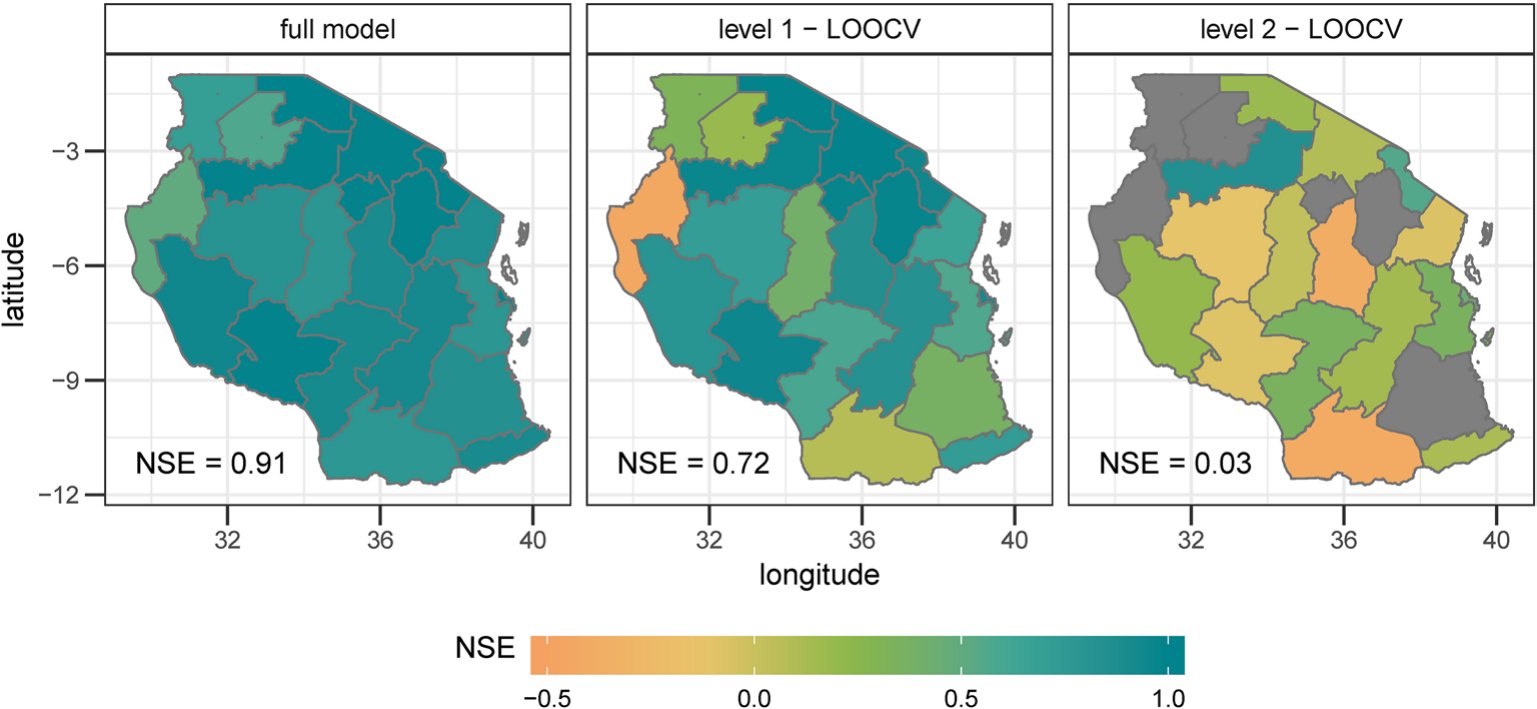
Performance of the forecast of anomalies based on selected region-specific weather and SST variables. Areas in grey show an NSE of lower than -0.5. Further explanation can be found under Fig. 2.

The forecast shows a high accuracy for the full model (NSE = 0.91) and the level 1 validation (NSE = 0.72). The results of the level 2 validation suggest that the variable selection has a high year-to-year variability so that the performance of this validation is lower. The level 2 validation has a high skill (NSE > 0.3) for 5 regions in Tanzania (Fig. 5). The middle panel of Fig. 8 shows the yearly performance of the forecast of anomalies.

We tested the performance of the forecast compared to a constant model that only takes the mean yield excluding the year that is forecasted as a predictor. The lower RMSE of the forecast compared to a constant model (SI Table 4) underlines the robustness of the forecasting results. A strong correlation between SST and weather variables exists only in few, non-systematic cases (SI Fig. 4) and is eliminated due to the applied filter during the variable selection. Thus a collinearity between SST and weather variables is not existent.

**Figure 6:**
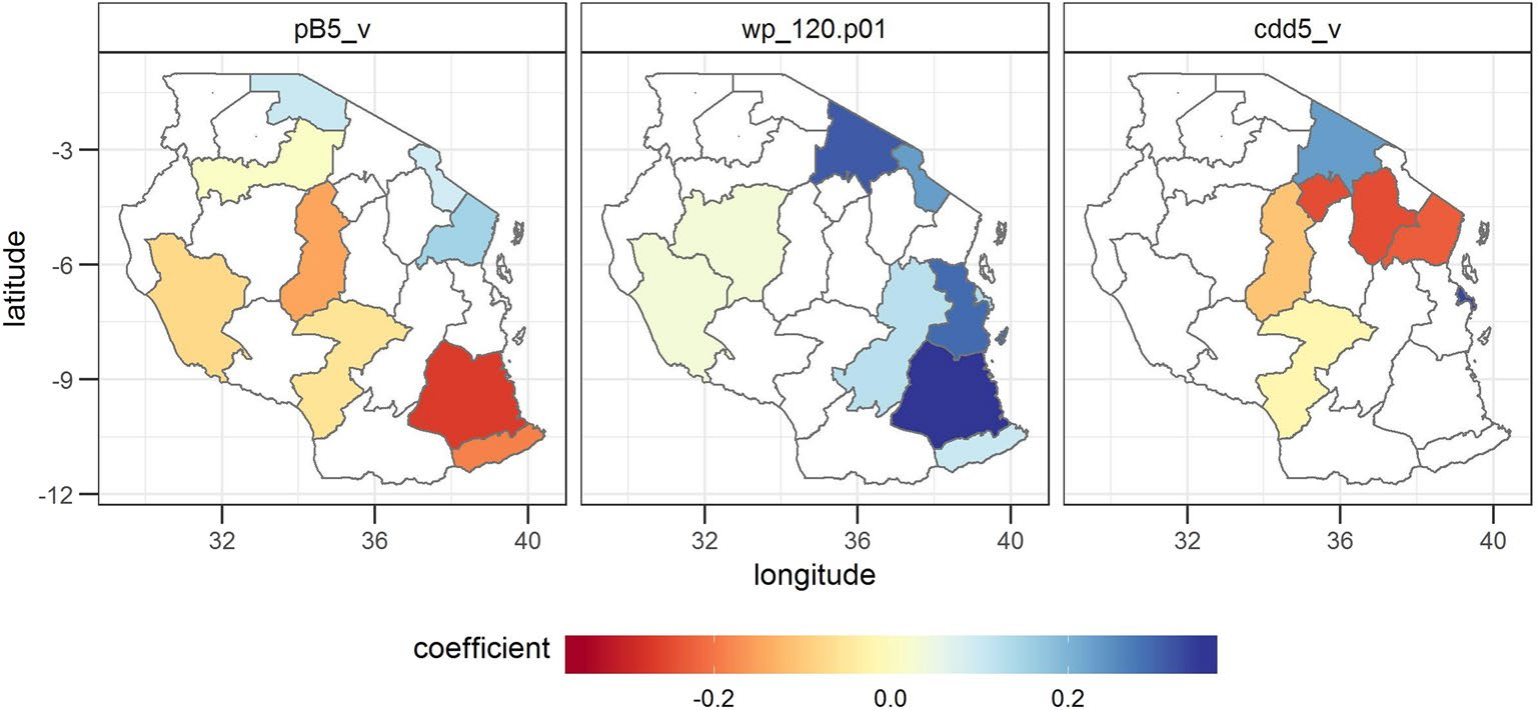
Coefficients of the three most often selected variables in the forecast based on weather and SST variables. Further explanations can be found under Fig. 3. The statistical significance of the estimated regression coefficients is shown in SI Fig. 3.

The variable selection of the forecast reveals similar variables compared to the model based on variables of the vegetative and the reproductive phase. The three most often selected variables are shown in Fig. 6. Precipitation events below 5 mm (pB5) are positively correlated with maize yields in the North, but negatively correlated with yields in the rest of the country. Whereas the negative correlation could indicate a negative influence of low precipitation events on yields due to insufficient total rainfall amounts in the vegetative phase, the positive correlation could indicate a positive influence of moderate and well distributed rainfall. The number of times the SST in WP falls below the 1% percentile with a lead time of 120 days (wp_120.p01) is positively correlated with yields, which could be explained by above average rainfall amounts in case of negative SST anomalies in the West Pacific. Dry conditions represented by the number of consecutive dry days of more than 5 days (cdd5) are mostly negatively correlated with yields.

### Forecasting absolute yields

As required for operational forecasting systems, we also provide a forecast of absolute yields. To obtain absolute yields, we added the previously subtracted trend and the mean yield to the forecasted yield anomalies.

**Figure 7:**
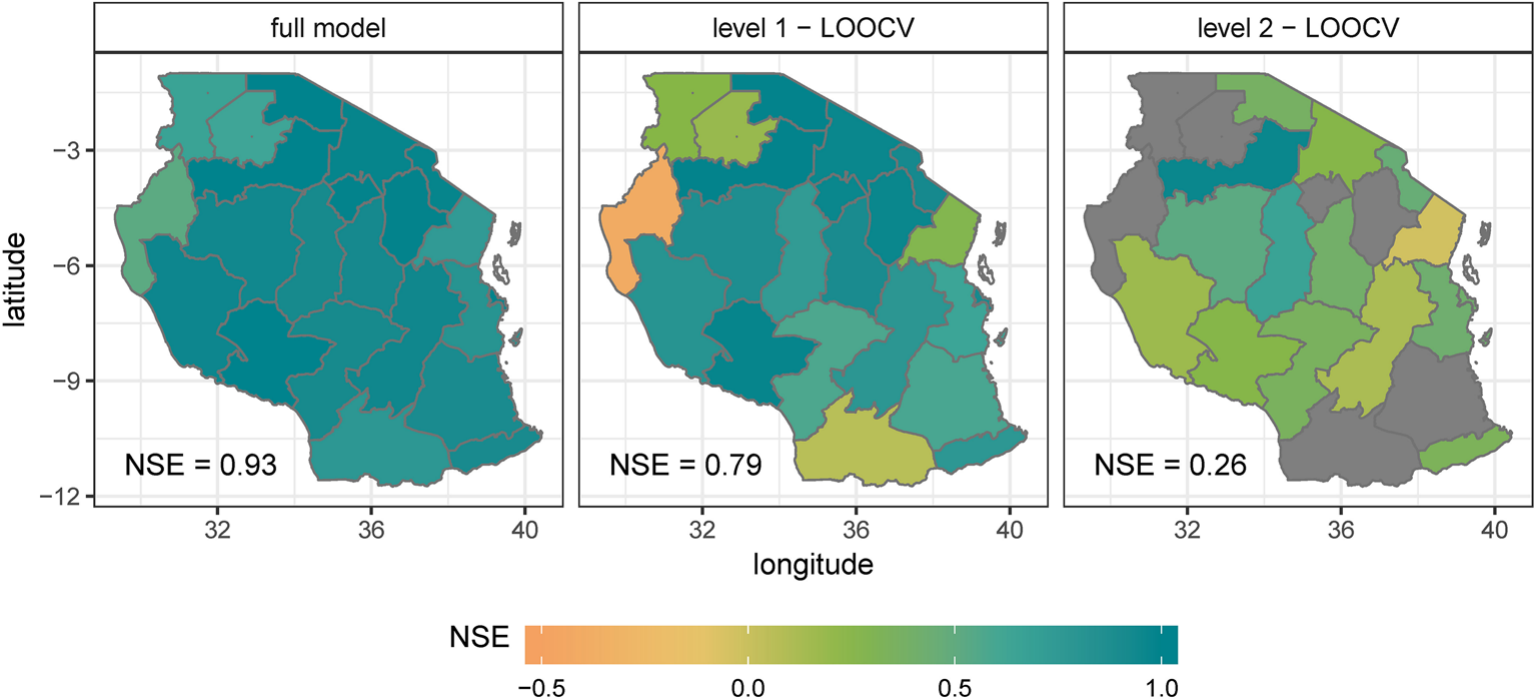
Assessment of the forecasted absolute yields. Areas in grey show an NSE of lower than − 0.5.

The full model evaluation and the level 1 validation indicate a high skill (median NSE of 0.93 and 0.79) of the within-season forecast of absolute yields for the whole country (Fig. 7). The level 2 validation has a median NSE of 0.26 and shows a high performance for 10 of 21 regions, which have an NSE higher than 0.3. The median RMSE is 0.07 t/ha for the estimation, 0.13 t/ha for the level 1 validation and 0.3 t/ha for the level 2 validation.

**Figure 8:**
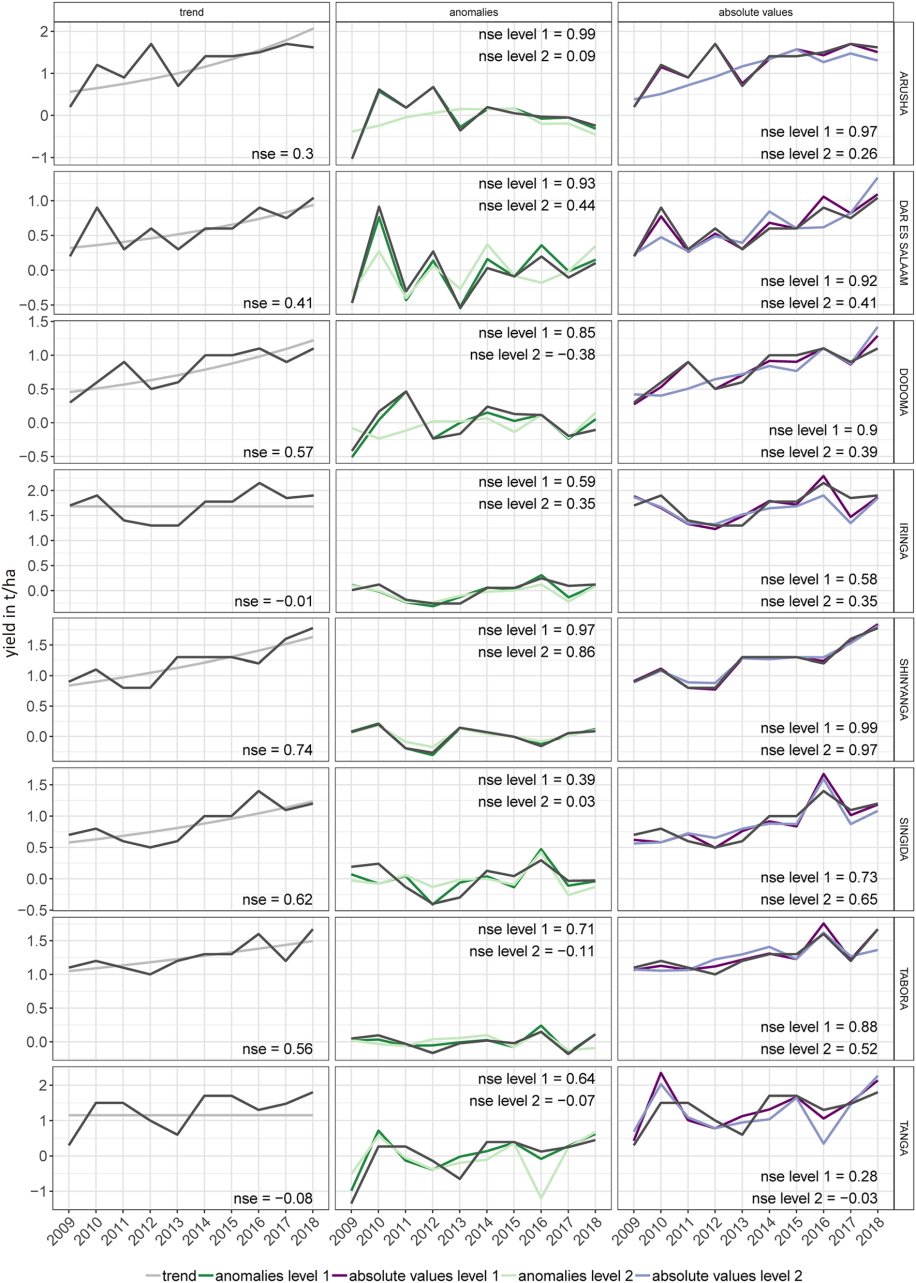
Regional performance of the forecasts derived from the model combining SST and weather inputs. Model assessment was done separately for the trend (left panel), the variability (middle panel) and absolute yields, which is a combination of trend and variability (right panel) for 8 of 21 regions in Tanzania. Plots for all 21 regions can be found in SI Fig. 5. The dark colour (dark green and dark purple) shows the forecast when the level 1 validation is applied. The corresponding NSE value is shown as ‘nse.l1'. The light colour (light green and light purple) and the ‘nse.l2' show the results of the level 2 validation. Because the trend was fitted based on logarithmic values, the transformation back to linear values results in a slightly curved shape.

The reason for the improved forecasting performance is, in most regions, due to the inclusion of the trend, in particular for the level 2 validation (Fig. 8).

### Application of the forecast for year 2019

**Figure 9:**
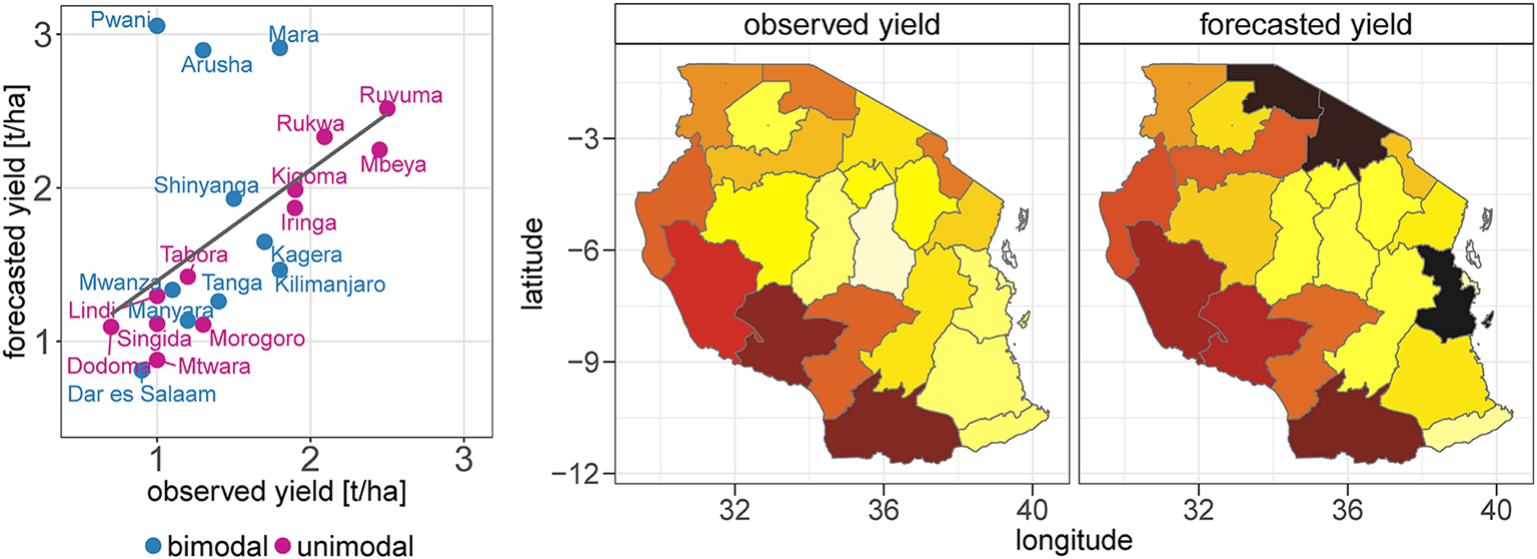
Assessments of the forecasted absolute yields for 2019 in regions with unimodal and bimodal rainfall regimes.

We used the model trained on yield data from 2009 to 2018 to provide a completely independent forecast for the harvest year 2019. The forecast of absolute yields for 2019 shows an overall high performance of the forecast in regions with a unimodal rainfall regime (NSE of 0.89 in the level 2—LOOCV) and a weak performance in bimodal rainfall regions (NSE of − 7.73 in the level 2—LOOCV; Fig. 9).

## Discussion

Our study provides a within-season maize yield forecast for entire Tanzania and is, to our best knowledge, the first of its kind. In contrast to existing forecasting studies in East Africa^2,3^, we provide an out-of-sample validation and an out-of-sample variable selection. Furthermore, we test the robustness by providing a completely independent forecast for the harvest year 2019. This strict and transparent assessment of uncertainties of the forecast is particularly important in practice, when e.g. policy makers use a yield forecast to inform food security planning.

Over all regions, the forecast of anomalies produces a median NSE of 0.91 in the model estimation and a median NSE of 0.72 in the level 1 validation, which indicates an accurate and robust forecast for large parts of the country. The forecast reproduces both extreme years as well as average yields with high accuracy at a lead time of on average 6 weeks.

Weather influences explain a substantial share of observed maize yield variability in Tanzania as shown by the national median NSE of 0.92 in the estimation and 0.81 in the level 1 validation of the yield model based on weather inputs. Precipitation as well as higher minimum temperatures positively influence maize yields. The variable *consecutive dry days* is the most often selected variable across the regions, which underlines the detrimental effect of dry weather conditions on maize yields. This is in line with Cairns et al. (2013)^6^ and Rowhani et al. (2011)^7^ who found drought stress to be one of the main weather-related reasons for low maize yields in Sub-Saharan Africa. Apart from weather inputs, the SST of the West Pacific (WP) provides most explained yield variability in the forecast. This can be explained by a strong correlation between the SST in the WP and East African rain^8^. Low SST in the WP, which are related with higher rainfall amounts during the long rains in East Africa^8–10^, are positively correlated with maize yields. The variable selection shows that maize yield variability in Tanzania is strongly influenced by extreme events. Also the study of Rowhani et al. (2011)^7^ focusing on climate variability and crop production in Tanzania seems to be in line with our finding. They found a strong relationship between the coefficient of variation (cv) of weather variables and cereal yields. In contrast to the cv, the percentile variables can explicitly represent lower or upper extremes and are therefore easier to interpret. The importance of extreme events for explaining maize yield variability in Tanzania implies that there is an upper quality bound for yield forecasts in case of extreme events that are out of the considered range of the training data. The variables selected in the estimation and the forecast show strong similarities for most regions, which suggests that the variable selection is robust.

In contrast to the high estimation and level 1 validation performance, the level 2 validation, which additionally integrates an out-of-sample variable selection, only shows good skills for a limited number of regions in Tanzania. This rigorous level 2 validation is most relevant in practice, as at the moment of forecasting (1–2 month before the harvest), the variable selection can only be done based on yield information from previous years. Despite its importance, this validation is rarely applied by forecasting studies and more than half of the studies even lack a level 1 validation^11^. The study of Ogutu et al. (2018)^3^ who provide a forecast for East Africa does not even assess the model results based on observational data. To guarantee that the weaker level 2 performance is not due to model overfitting for the short time series at our disposal, we used a precautionary approach of only allowing a maximum of 5 variables to be selected for a time series of 10 years. This leaves sufficient degrees of freedom (5 DF in the full model and 4 DF for the validation) so that we assume model overfitting to the training as not the main reason for lower performance. Instead, the weaker level 2 performance could be related to the following reasons. First, the short length of the time series of only 10 years may not be sufficient to provide a stable out-of-sample variable selection. In our study period, several ENSO events (e.g. El Niño in 2015/2016 or 2009/2010 and La Nina in 2010/2011 or 2011/2012^12^) and Indian Ocean Dipole phases (e.g. positive phases in 2019 and 2015 and negative phases in 2016 and 2010^13^) occurred, which bring high year-to-year variation in weather influences and consequently in the variable selection. The time series may not be long enough to train the model for this highly variable weather. The forecast should be repeated and tested on a longer time series when more data becomes available. Also the prediction of ENSO Events^14,15^ can be used to inform the yield forecast. Second, uncertainties in the calculated start and end of the growing season, as well as the calculated separation between the vegetative and the reproductive phase can lead to a lower performance of the forecast or inaccuracies in the lead time. The calculated dates may not reflect the real phenological phases and the static dates do not account for the inner-seasonal variability due to differing weather conditions. Third, the forecast relies on reported yield data, which is prone to reporting errors and data quality issues, which may change the forecasting performance.

Because of its relevance in practice, we also provide a forecast of absolute yields, which in addition to the yield variability also includes information about the trend. The results of the absolute yield forecasts are slightly better (median NSE of 0.79 in the level 1 validation) due to the explanatory power of the trend. The disentangling of trend and variability provides transparency about the reasons for the forecasting skills so that in case of a weak weather signal, the trend can be used as the best available information about the expected yields. The forecast is only valid when assuming that the trend of the previous years is going to continue for the following years. For grounding such an assumption, a causal analysis of trends taking into account national policies^16^ and international market conditions, as it has been examined for East African countries^17^ and the Usangu plain in Tanzania^18^, would be mandatory.

The completely independent forecast of yields for the harvest year 2019 shows a high performance for the unimodal rainfall areas and a poor performance in bimodal rainfall regions. The poor performance in the bimodal regions could be related to the outbreak of the fall armyworms, which particularly hit the bimodal rainfall areas in northern, north-eastern and the coastal areas. The below average rainfall during the long rains in these areas fostered the outbreak of the fall armyworms so that an infestation level of 50% was reached in some regions^19^. This result underlines the importance of incorporating local expert knowledge in the evaluation of an operational forecast. Yields in some regions may be strongly influenced by factors that the model does not account for, such as pests and diseases or political, economic or social changes. Therefore, the forecast—as a tool that provides quantitative information about the expected weather-related yields—should be embedded in a forecasting system that integrates several sources of information for the evaluation of the food security situation.

In this study, we constructed an empirical model to decipher climatic influences on maize yield variation in Tanzania. We applied this model to forecast maize yield anomalies and absolute yields at a lead time of about 6 weeks before the harvest on region level in Tanzania. The model provides accurate and robust yield forecasts for large parts of the country. The strict and transparent assessment of the forecasting skill and the low requirements of input data make the forecast potentially suitable to inform operational yield forecasting systems in other countries with inaccessible or low quality weather data and short time series yield data. The proposed model can contribute to better informed local agronomic management strategies and support the implementation of regional agricultural development programs so that food shortages caused by unfavourable weather conditions can be mitigated. This has the potential to accelerate investments in these programs and thus, reduce food insecurity.

## Methods

### Yield data

We used official maize yield data from 2009 to 2019 obtained from the National Food Security Division of the Ministry of Agriculture of Tanzania. Maize is the most important staple crop in Tanzania^4^ and had a total harvest area of 3,428,630 ha in 2019, which represents ca. 25% of the total crop land in Tanzania^20^ (statistics from 2017). Maize yields in Tanzania show a high variability and were on average at 1.6 t/ha from 2016 to 2018 (SI Fig. 6). The official statistics report yearly yield data covering all 21 regions of Mainland Tanzania (excluding the island Zanzibar). The administrative boundaries of some regions changed in 2012. To obtain a consistent time series, we used the administrative boundaries from before 2012 by applying a weighted average based on area.

### Weather data

We created weather variables based on two climate data sources. For precipitation, we used observed daily rainfall totals from CHIRPS (Climate Hazards group Infrared Precipitation with Stations) at a resolution of 0.25° × 0.25°^21^. CHIRPS provides reliable precipitation information for East Africa and outperforms previous products, like ARC2 and TAMSAT^22,23^. For daily mean, maximum and minimum temperature, we used ERA5 data^24^ provided at a spatial resolution of 0.25° × 0.25°. ERA5 is the most recent reanalyses product and outperforms ERA-Interim for East Africa^25^. The weather variables were created for the time period of available yield statistics (2009–2019), including weather data for the year 2008 in case of growing seasons starting already at the end of 2008.

### SST data

In addition to weather data, we included monthly sea surface temperature (SST) anomalies^26^ of (1) the El Niño 3.4 zone (170°W–120°W, 5°S–5°N), (2) the West Pacific Box (WP) (130°E–160°E, 10°S–10°N) and (3) the Indian Ocean Dipole (IOD), which is the non-normalized difference between the West Indian Ocean (50°E–70°E, 10°S–10°N) and the Eastern Indian Ocean (90°E–110°E, 10°S–0°N). We included these SST indicators due to their documented influence on East African rainfall. SST anomalies in the El Niño 3.4 zone and the IOD have shown to be positively correlated with rainfall over East Africa during the short rains, whereas SST anomalies in the WP show a negative correlation with East African rainfall during the long rains^8–10^. The influence of these indicators on East African precipitation has a lag of some weeks to months offering the potential to provide yield forecasts at longer lead times. We tested different lead times and included those that showed the highest correlation with yield (SI Fig. 7), i.e. a lead time of 120 days for El Niño 3.4 and WP; and a lead time of 30 days for IOD.

### Growing season

The North, North-East and the coastal areas of Tanzania are characterized by a bimodal rainfall pattern. The so-called ‘short rains’ (or Vuli) occur from October to December and the ‘long rains’ (Masika) last from March to May. The rest of the country has a unimodal rainfall pattern called Musumi with rainfall occurring from December to April^9^. Because of the low availability of irrigation facilities in Tanzania^20^, we did not distinguish irrigated from non-irrigated agriculture and considered the growing seasons to be aligned with the onset of the rain.

We defined the start of the growing season following the approach of Dodd and Jolliffe (2001)^27^, which was tested for tropical and subtropical conditions. They define the onset of the growing season when the following three criteria are fulfilled:

1. at least 25 mm rainfall within 6 days
2. starting day and at least 2 other days in this 6-day period are wet (> 0.1 mm)
3. no dry period of 10 or more consecutive days within next 40 days

Because of the bimodal rainfall pattern in North and North-East Tanzania, two onsets of the growing season are found for some grid points. In this case, we considered the onset of the long rains (Masika), which is the main growing season. We used a static crop calendar at region level by first calculating the onset and the end of the growing season per grid point for each harvest year and then calculating the median over all grid points within a region and then a median over all years. We also tested other approaches (SI Section 8) to test the robustness of the results.

We defined the end of the growing season as 110 days after the start, which corresponds to the average time from sowing to maturity of 4 typical maize cultivars in Tanzania (i.e. Stuka, Staha, TMV1 and Pioneer HB3253). This is a reasonable choice since individual growing season lengths vary in a small range from 105 to 114 days^28^.

### Model inputs

Based on temperature and precipitation data, we created variables that account for the climate drivers that influence maize development and yields (SI Tables 1, 2, 3 show a list of model inputs).

In addition to the median daily mean, maximum and minimum temperature over the growing season (*tas.median, tas.max, tas.min*), we included variables related to extreme temperatures. Above the optimum temperature range, photosynthesis is reduced, whereas respiration rates rise, such that net photosynthesis rates decline^29^. Particularly during flowering, maize is sensitive to heat stress, as it can lead to the desiccation of pollen or a reduction in grain numbers. High temperatures accelerate the development rate and result in a shortened growing season and thus a reduction in light perception. Particularly if the time for grain filling is reduced, grain size and consequently yields decline^30^. To account for the damaging impacts of too high temperatures, we included the number of days where the daily maximum temperature exceeds the region-specific long term 99% percentile of the maximum temperature in the growing season (*tas.max99;* SI Section 9 Eq. 1). Also temperatures below the optimum temperature range, i.e. any temperature below 25 °C according to Rötter and Van De Geijn (1999)^31^, are detrimental for maize growth. Therefore, we include the number of times the daily minimum temperature falls below the region-specific long-term 1% percentile of the minimum temperature (*tas.min01*).

The optimal rainfall required in the growing season for maize is around 500–800 mm^32^. We included the precipitation sum (*Psum*) in the growing season to represent the overall water availability for maize. For optimal plant development, the timing and duration of water supply are equally critical. During flowering, maize requires sufficient water supply. Before pollination water stress leads to kernel abortion, even if at the time of pollination sufficient water is available. Excessive rain, in contrast, can lead to soil water saturation and oxygen deficiency, which limits root respiration and the growth of roots. Rainfall that exceeds the water holding capacity of the soil can lead to the leaching of nutrients and nutrient deficiencies of the plant^33^. To represent different precipitation ranges, we included the number of days with precipitation above a threshold of 5, 10 and 15 mm (*pA5*, *pA10*, *pA15*, respectively) and below a threshold of 5, 10 and 15 mm (*pB5, pB10, pB15*, respectively). Because the distribution of rainfall within the growing season is of particular importance for plant growth, we included consecutive dry days of more than 5, 10, 15 and 20 days (*cdd5, cdd10, cdd15*, *cdd20*, respectively). Extremely high precipitation events are covered by the number of times the daily precipitation sum exceeds the region-specific long-term 99% percentile of the daily precipitation sum.

The variables were separately calculated for the vegetative and reproductive phase of the growing season. The separation between both phases was based on the sum of growing degree days (GDD; SI Section 9 Eq. 2). The days in the growing season until 50% of the full-season GDD sum was reached were allocated to the vegetative phase and the remainder to the reproductive phase, following Schauberger et al. (2017)^34^.

The SST temperatures were aggregated by taking the median over the growing season. We also included the number of times the 99% percentile of the whole time series is exceeded and the number of times the indicator is below the 1% percentile as an additional variable. The SST indices are only created for the vegetative phase (including the lead time) based on the same approach as for the weather variables.

### Model development and assessment

**Figure 10:**
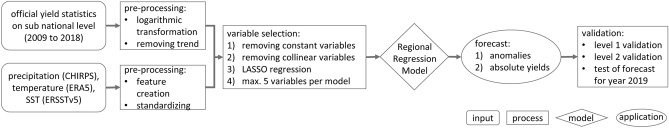
Modelling flow chart.

For each of the 21 regions, we applied the work flow as shown in Fig. 10, i.e. we provide the forecast separately for each region comprising of region-specific variables and parameters to account for the diverse climatic conditions within the country.

For our analysis, we used *R*^35^ (version 3.5.0) with the packages *tidyr*^36^ and *plyr*^37^ for data pre-processing, the packages *sp*^38^ and *rgdal*^39^ for spatial data processing, the package *glmnet*^40^ to perform LASSO regression and the package *ggplot2* ^41^ to generate the figures and maps.

#### Pre-processing

We standardized all weather and SST input data to allow for a better comparability of the beta coefficients^42^. We transformed the yield input data to logarithmic values and removed the trend by first testing different de-trending methods (mean, linear, quadratic) and then applying the one that resulted in the lowest Akaike Information Criterion (AIC).

#### Variable selection

We applied the following variable selection process to elucidate important variables for explaining yield variability in the different regions:

- We removed variables that do not show year-to-year variations (i.e. zero variance).
- To avoid multicollinearity, only those variables were selected that are not strongly collinear (i.e. Pearson’s r > 0.7) with another explanatory variable. If a pair of variables was strongly collinear, then the variable with the higher correlation with yield anomalies was selected.
- Input selection was done using LASSO regression. Through regularization, LASSO performs a co-variate selection, which improves both the prediction accuracy and the interpretability^43^. To select the optimum lambda (the regularization penalty for the LASSO regression), we used the lowest cross-validation (years were omitted subsequently) mean squared error (MSE).
- At last, we removed all variables except for those 5 variables showing the highest correlation with yields. This step was conducted to reduce overfitting of the model based on the rule-of-thumb to include a maximum of half as many independent variables (climate variables) as there are dependent variables (yields).

#### Model for estimating and forecasting yields before harvest

For each region, we applied a separate regional regression model (Eq. 1) following the approach of Gornott and Wechsung (2016)^44^ and Schauberger et al. (2017)^34^.

1 $${\text{log}}(y_{it} ) = \mathop \sum \limits_{k = 1}^{K} \beta_{ki} x_{kit} + \varepsilon_{it }$$

with $\beta$ as parameters, $y$ as the demeaned and detrended response variable, $x$ as the standardized explanatory input variable, $\varepsilon$ as error term for $K$ variables ($k = 1, \ldots ,K$), $N$ spatial units ($i = 1, \ldots ,N$) and $T$ years ($t = 1, \ldots ,T$).

With the Breusch–Godfrey and the Breusch–Pagan tests we tested autocorrelation and heteroscedasticity of model residuals. We use the Variance Inflation Factor to test for multicolinearity.

For the within-season forecast, we only included variables during the vegetative phase. This provides a lead time of around 55 days, i.e. around 6 weeks before the harvest in a growing season totalling 110 days. The exact lead time differs per region—depending on the start of the reproductive phase in each region (SI Table 5). We provided the forecast for yield anomalies (i.e. variation around a trend) and for absolute values. For the latter, we added the trend and retransformed the values back to the linear form.

#### Cross validation

We applied a two level leave-one-out cross validation (LOOCV).

Level 1—LOOCV: we selected the variables based on all observations with LASSO. Then we subsequently removed observations for 1 year from the data set and used the remaining observations from the other years to fit the model and predict yield changes for the removed year, using the pre-selected set of variables.

Level 2—LOOCV: we subsequently removed observations for 1 year from the data set, then selected variables with LASSO and estimated the model based on the remaining data set. We used this model to predict yield changes for the removed year. This level 2—LOOCV guarantees that no information from the removed year is used for the variable selection or the model estimation. For this validation we allow a maximum of only 4 variables per model (allowing one less than half as many variables as there are observations). This validation simulates the operational context, where no yield information from the year to be forecasted is available for model building.

In addition, we used the model we developed based on the yield data from 2009 to 2018 to provide a completely independent forecast for the harvest year 2019. The yield data for 2019 was not known to us during the model development.

The goodness of fit between the observed data and the predicted data was then evaluated based on the Nash–Sutcliffe efficiency coefficient^5^ (NSE). In contrast to the explained variance (r^2^), NSE does not only evaluate similarities in variability, but also integrates the mean model bias, which makes it a robust measure of the prediction quality. We also calculated the root mean squared error (RMSE) to estimate the average error between observed and predicted yields.

## Supplementary information

Supplementary Information.

## Data Availability

All data supporting the findings of this study are either public data sets, are available within the article and its Supplementary Information files or are available from the corresponding author upon reasonable request.
