# Supplementary material for: Robustly forecasting maize yields in Tanzania based on climatic predictors
Source: Sci Rep. 2020 Nov 12;10:19650. doi: 10.1038/s41598-020-76315-8 (PMC7665066; doi:10.1038/s41598-020-76315-8)
Supplement: Supplementary file 1 — Supplementary Information. [file 41598_2020_76315_MOESM1_ESM.docx]

Supplementary Information to “Robustly forecasting maize yields in Tanzania based on climatic predictors”

**Author names and affiliations:**

Rahel Laudien^a*^, Bernhard Schauberger^a^, David Makowski^b^, Christoph Gornott^ac^

^a^Potsdam Institute for Climate Impact Research (PIK)

Member of the Leibniz Association
P.O. Box 60 12 03
D-14412 Potsdam
Germany

^b^National Research Institute for Agriculture, Food and Environment (INRAE)

UMR 518 AgroParisTech Université Paris-Saclay

16 rue Claude Bernard

F-75231 Paris Cedex 05

France

^c^Agroecosystem Analysis and Modelling

Faculty of Organic Agricultural Sciences

University of Kassel

Mönchebergstraße 19

34109 Kassel

Germany

**Corresponding author:**

Rahel Laudien ([laudien@pik-potsdam.de](mailto:laudien@pik-potsdam.de), +49 331 28820771)

**Co-authors:**

Christoph Gornott ([gornott@pik-potsdam.de](mailto:gornott@pik-potsdam.de), +49 331 2882655)

Bernhard Schauberger ([schauber@pik-potsdam.de](mailto:schauber@pik-potsdam.de), +49 331 28820890)

David Makowski ([david.makowski@inrae.fr](mailto:david.makowski@inrae.fr), +33 698883675)

# Input variables for the Regional Regression Model

| input name | Definition | unit | Vegetative phase | | | Reproductive phase | | |
| --- | --- | --- | --- | --- | --- | --- | --- | --- |
|  |  |  | median | min | max | median | min | max |
| psum | Precipitation sum | mm | 303.97 | 85.50 | 1080.59 | 200.86 | 3.65 | 689.90 |
| cdd5 | Consecutive dry days of equal or more than 5 days |  | 1.00 | 0.00 | 4.00 | 2.00 | 0.00 | 6.00 |
| cdd10 | Consecutive dry days of equal or more than 10 days |  | 0.00 | 0.00 | 2.00 | 0.00 | 0.00 | 3.00 |
| cdd15 | Consecutive dry days of equal or more than 15 days |  | 0.00 | 0.00 | 1.00 | 0.00 | 0.00 | 2.00 |
| cdd20 | Consecutive dry days of equal or more than 20 days |  | 0.00 | 0.00 | 1.00 | 0.00 | 0.00 | 1.00 |
| pB5 | Number of precipitation events below 5mm per day |  | 9.00 | 3.00 | 19.00 | 8.00 | 2.00 | 25.00 |
| pB10 | Number of precipitation events below 10mm per day |  | 18.00 | 6.00 | 35.00 | 13.00 | 2.00 | 34.00 |
| pB15 | Number of precipitation events below 15mm per day |  | 23.50 | 8.00 | 40.00 | 17.00 | 2.00 | 38.00 |
| pA5 | Number of precipitation events equal or above 5mm per day |  | 20.00 | 6.00 | 39.00 | 14.00 | 0.00 | 33.00 |
| pA10 | Number of precipitation events equal or above 10mm per day |  | 11.00 | 2.00 | 26.00 | 7.00 | 0.00 | 25.00 |
| pA15 | Number of precipitation events equal or above 15mm per day |  | 6.00 | 0.00 | 18.00 | 3.00 | 0.00 | 18.00 |
| precip.p99 | Number of times the daily precipitation sum exceeds the 99% percentile of the daily precipitation sum |  | 0.00 | 0.00 | 4.00 | 0.00 | 0.00 | 3.00 |

**SI Table 1.** Variables related to precipitation

| input name | Definition | unit | Vegetative phase | | | Reproductive phase | | |
| --- | --- | --- | --- | --- | --- | --- | --- | --- |
|  |  |  | median | min | max | median | min | max |
| tas.median | Median of the daily mean temperature | °C | 23.93 | 21.51 | 28.18 | 23.87 | 19.81 | 27.42 |
| tas.max | Median of the daily maximum temperature | °C | 28.00 | 24.97 | 33.36 | 27.90 | 24.61 | 31.47 |
| tas.min | Median of the daily minimum temperature | °C | 19.29 | 17.58 | 26.28 | 19.21 | 14.34 | 25.22 |
| tas.max.p99 | Number of times the daily maximum temperature exceeds the 99% percentile of the daily maximum temperature |  | 0.00 | 0.00 | 9.00 | 0.00 | 0.00 | 7.00 |
| tas.min.p01 | Number of times the daily minimum temperature falls below the 1% percentile of the daily minimum temperature |  | 0.00 | 0.00 | 5.50 | 0.00 | 0.00 | 5.50 |

**SI Table 2.** Variables related to temperature

| input name | Definition | median | min | max |
| --- | --- | --- | --- | --- |
| iod_30.median | Median Indian Ocean Dipole over the last 30 days before the start of the growing season | -0.04 | -0.57 | 0.52 |
| iod_30.p01 | Number of times the Indian Ocean Dipole falls below the 1% Percentile of the Indian Ocean Dipole over the last 30 days before the start of the growing season | 0.00 | 0.00 | 2.00 |
| iod_30.p99 | Number of times the Indian Ocean Dipole exceeds the 99% Percentile of the Indian Ocean Dipole over the last 30 days before the start of the growing season | 3.00 | 1.00 | 3.00 |
| nino34_90.median | Median SST anomaly in the El Nino 3.4. zone over the last 90 days before the start of the growing season | -0.26 | -1.61 | 2.58 |
| nino34_90.p01 | Number of times the SST anomaly in the El Nino 3.4 falls below the 1% Percentile of the SST anomaly in the El Nino 3.4 zone over the last 90 days before the start of the growing season | 0.00 | 0.00 | 4.00 |
| nino34_90.p99 | Number of times the SST anomaly in the El Nino 3.4 zone exceeds the 99% Percentile of the SST anomaly in the El Nino 3.4 zone over the last 90 days before the start of the growing season | 5.00 | 0.00 | 5.00 |
| wp_90.median | Median SST anomaly in the West Pacific over that last 90 days before the start of the growing season | 0.42 | 0.08 | 0.85 |
| wp_90.p01 | Number of times the SST anomaly in the West Pacific falls below the 1% Percentile of the SST anomaly in the West Pacific over the last 90 days before the start of the growing season | 0.00 | 0.00 | 2.00 |
| wp_90.p99 | Number of times the SST anomaly in the West Pacific exceeds the 99% Percentile of the SST anomaly in the West Pacific over the last 90 days before the start of the growing season | 3.00 | 0.00 | 5.00 |

**SI Table 3.** Variables related to sea surface temperatures (SST)

# Significance of estimated regression coefficients


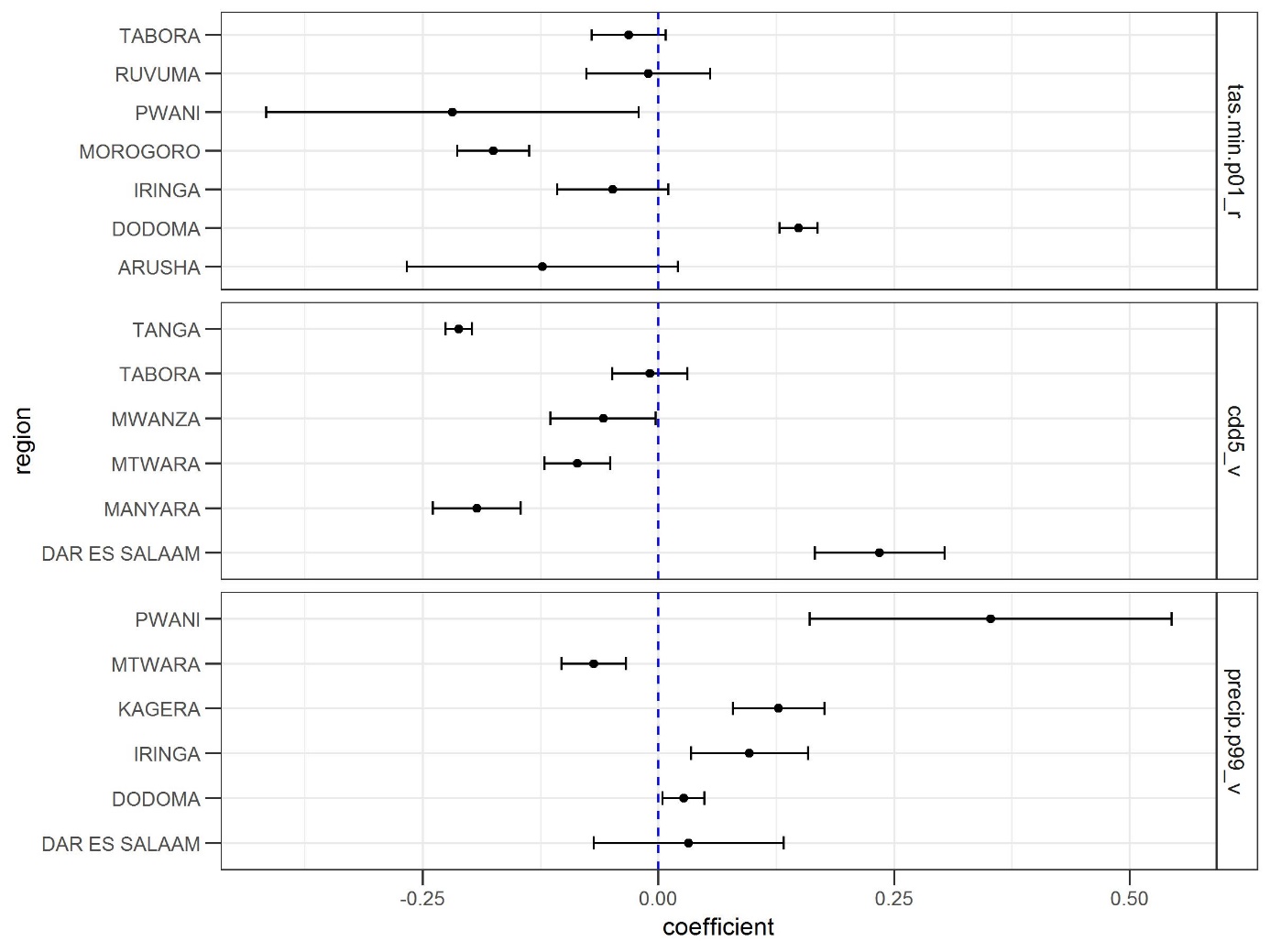


**SI Fig. 1.** Estimated regression coefficients for the three most often selected weather variables for Tanzanian regions. The three most often selected variables are temperature events below the 1% minimum temperature percentile in the reproductive period (tas.min.p01_r), consecutive dry days of more than 5 days in the vegetative phase (cdd5_v) and precipitation events above the 99th precipitation percentile in the vegetative phase (precip.p99_v). Note that this analysis excludes the model coefficients with a performance lower than an NSE of 0.3 in the level 1 validation, because we assumed these models as not robust enough for further analysis. The coefficients show standardised values, i.e. they show the change in yield per standard deviation of the input variable. The horizontal bars show the 95% significance interval for the point estimator. The geographic location of the regions can be seen in SI Fig. 6.


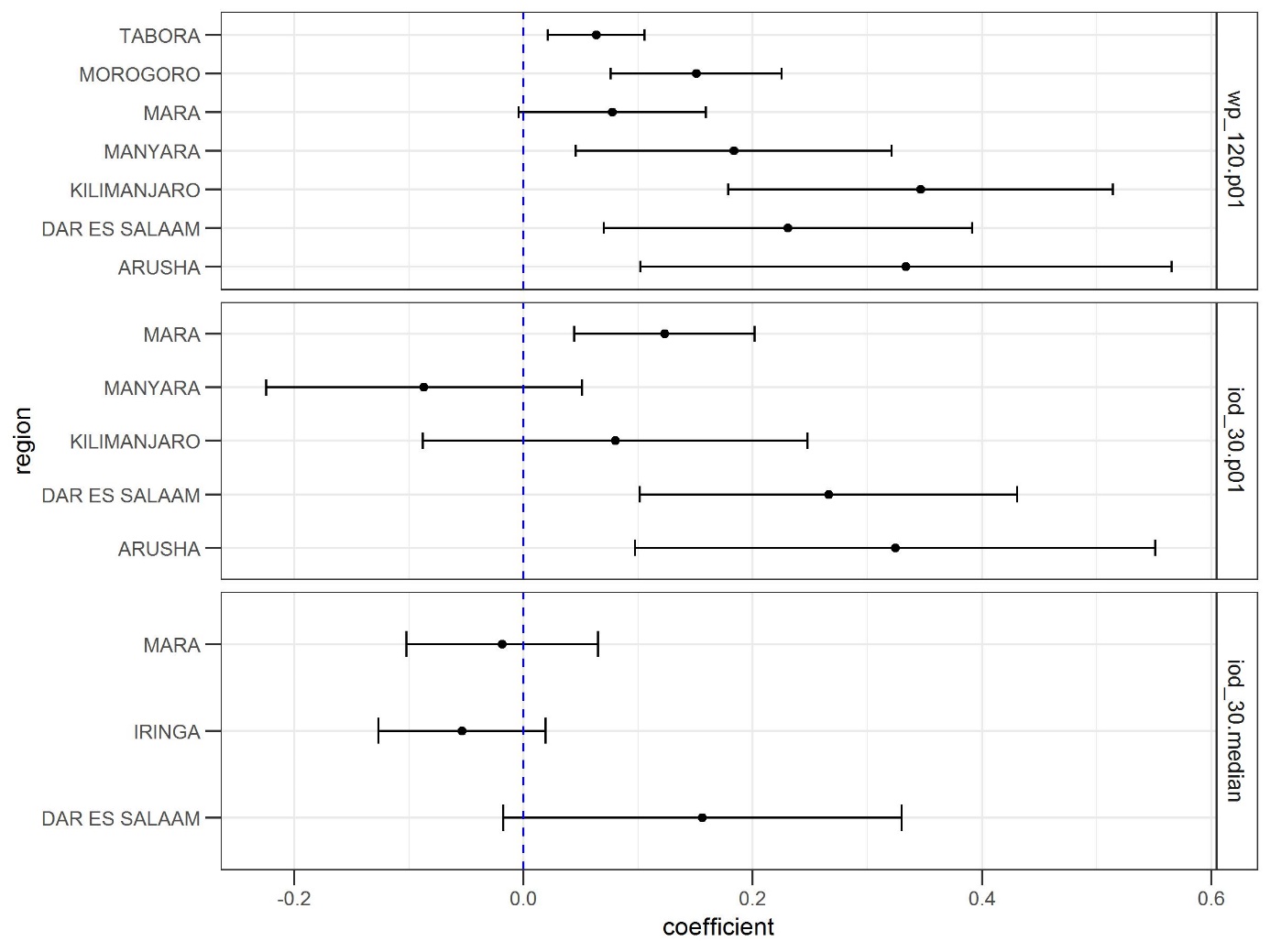


**SI Fig. 2.** Estimated regression coefficients for the three most often selected sea surface temperature (SST) variables for Tanzanian regions. The three most often selected variables are the number of times the SST falls below the 1% percentile of the West Pacific considering a lead time of 120 days (wp_120.p01), the number of times the SST falls below the 1% percentile of the Indian Ocean Dipole considering a lead time of 30 days (iod_30.p01) and the median SST of the IOD considering a lead time of 30 days (iod_30.median). Note that this analysis excludes the model coefficients with a performance lower than an NSE of 0.3 in the level 1 validation, because we assumed these models as not robust enough for further analysis. The coefficients show standardised values, i.e. they show the change in yield per standard deviation of the input variable. The horizontal bars show the 95% significance interval for the point estimator. The geographic location of the regions can be seen in SI Fig. 6.


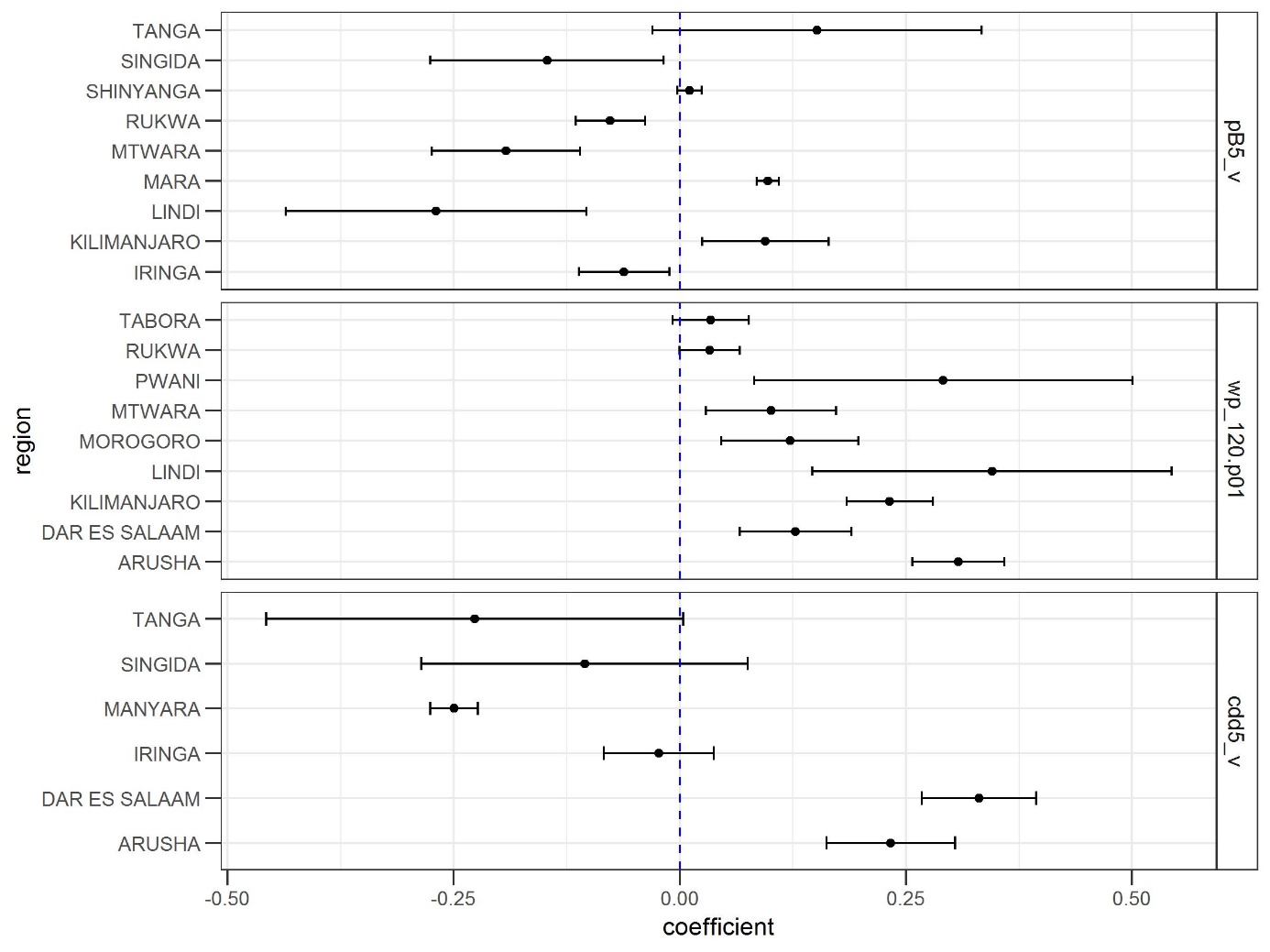


**SI Fig. 3.** Estimated regression coefficients for the three most often selected variables in the forecast based on weather and sea surface temperature (SST) variables for Tanzanian regions. The three most often selected variables are precipitation events below 5 mm in the vegetative phase (pB5_v), the number of times the SST in the West Pacific falls below the 1% percentile considering a lead time of 120 days (wp_120.p01) and consecutive dry days of more than 5 days in the vegetative phase (cdd5_v). Note that this analysis excludes the model coefficients with a performance lower than an NSE of 0.3 in the level 1 validation, because we assumed these models as not robust enough for further analysis. The coefficients show standardised values, i.e. they show the change in yield per standard deviation of the input variable. The horizontal bars show the 95% significance interval for the point estimator. The geographic location of the regions can be seen in SI Fig. 6.

# Comparison of forecasted anomalies to a constant model

| region | RMSE constant model | RMSE forecasted anomalies |
| --- | --- | --- |
| Dodoma | 0.28 | 0.08 |
| Arusha | 0.52 | 0.05 |
| Kilimanjaro | 0.44 | 0.04 |
| Tanga | 0.61 | 0.20 |
| Morogoro | 0.30 | 0.07 |
| Pwani | 0.58 | 0.24 |
| Dar es Salaam | 0.44 | 0.05 |
| Lindi | 0.30 | 0.11 |
| Mtwara | 0.32 | 0.09 |
| Ruvuma | 0.12 | 0.05 |
| Iringa | 0.18 | 0.05 |
| Mbeya | 0.13 | 0.01 |
| Singida | 0.24 | 0.10 |
| Tabora | 0.12 | 0.04 |
| Rukwa | 0.14 | 0.03 |
| Kigoma | 0.10 | 0.06 |
| Shinyanga | 0.16 | 0.01 |
| Kagera | 0.20 | 0.10 |
| Mwanza | 0.13 | 0.07 |
| Mara | 0.19 | 0.01 |
| Manyara | 0.31 | 0.03 |

**SI Table 4.** Comparison of the performance of the forecasted yield anomalies with a lead time of ca. 6 weeks (right column) and a constant model that only takes the mean yield excluding the year that is forecasted as a predictor (middle column). The comparison is based on the root mean squared error (RMSE) between the observed yield and the modelled yield.

# Correlation between input variables


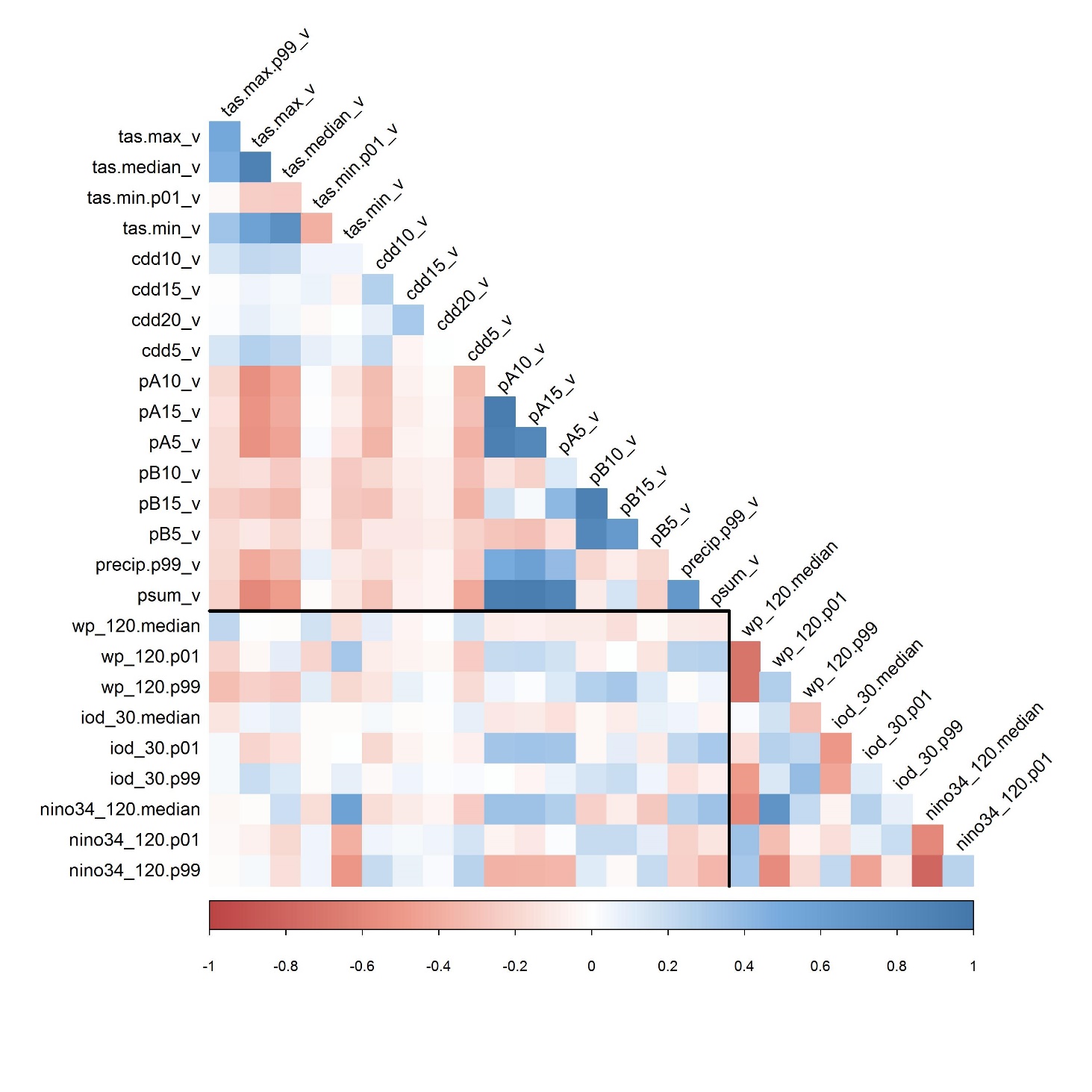


**SI Fig. 4.** Correlogram of input variables in sea surface temperature (SST) and weather categories; a strong correlation (absolute value of Pearson's r coefficient > 0.5) between SST and weather variables can only be found between the minimum temperature and the median SST in the El Niño 3.4 zone with a lead time of 120 days.

# Performance of the forecasts derived from the model combining SST and weather inputs


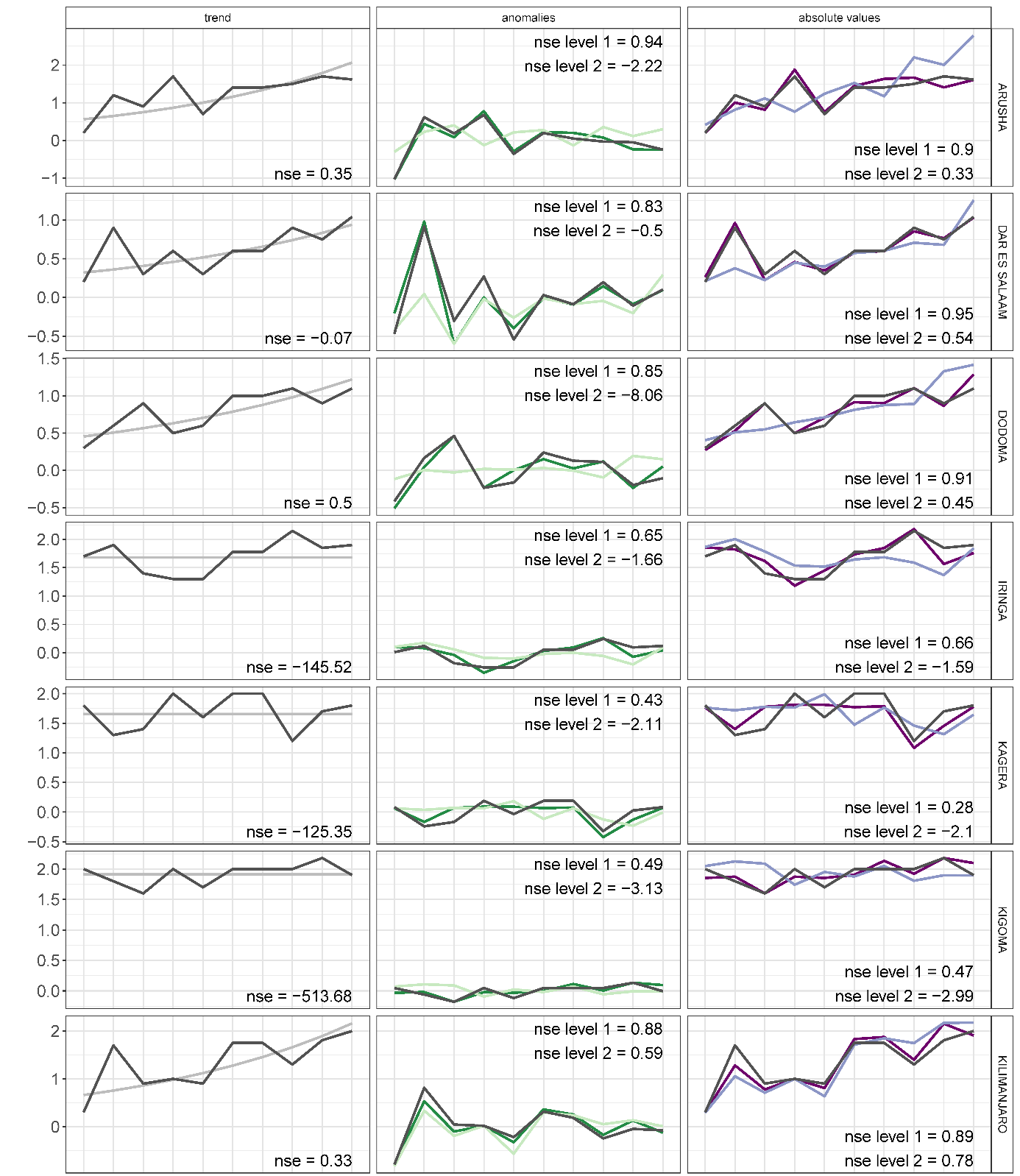


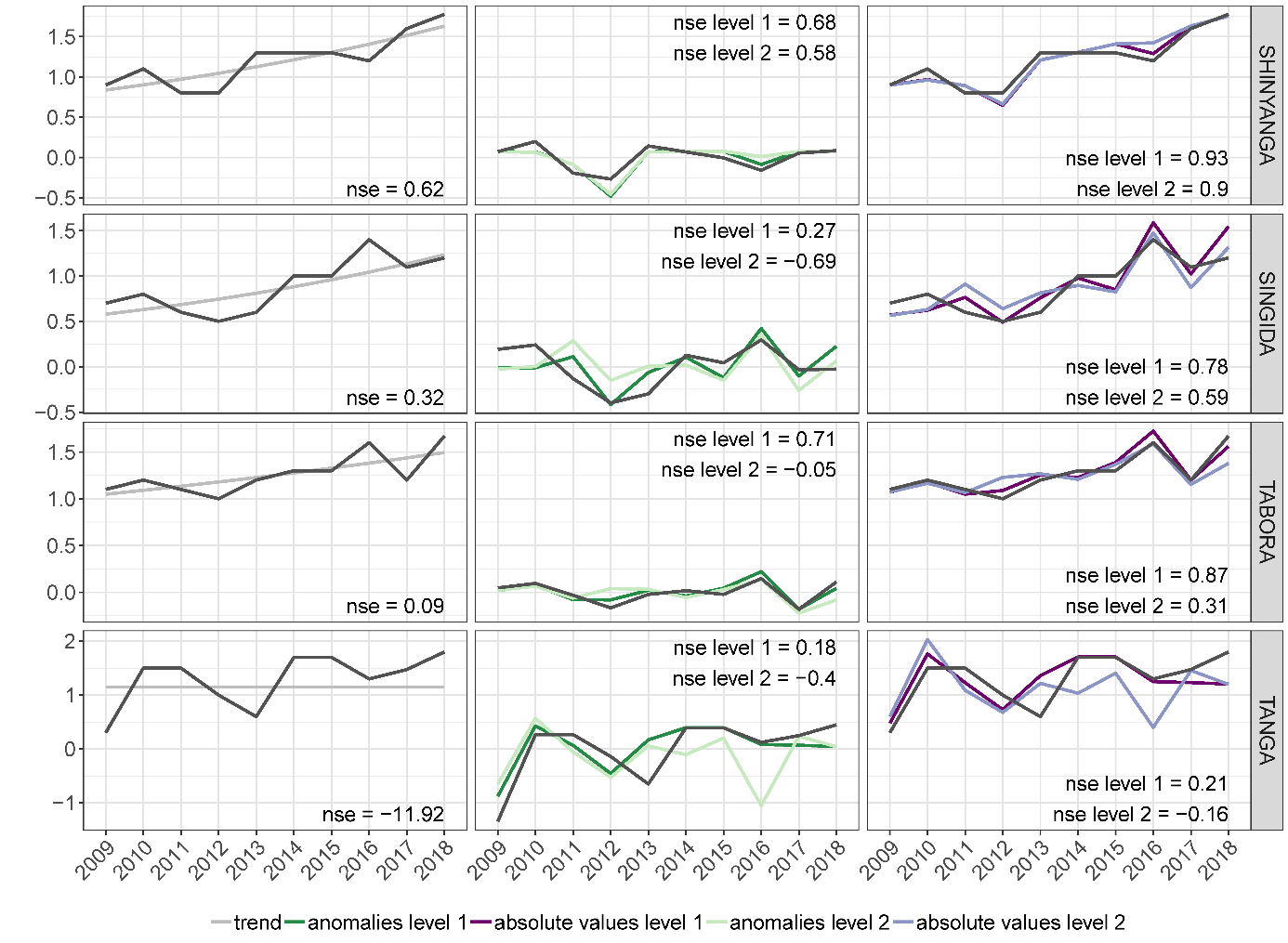


**SI Fig. 5.** Regional performance of the forecasts derived from the model combining SST and weather inputs. Model assessment was done separately for the trend (left panel), the variability (middle panel) and the absolute yields, which is a combination of trend and variability (right panel). The dark colour (dark green and dark purple) shows the forecast when the level 1 validation is applied. The corresponding NSE value is shown as “nse level 1” in the upper right corner of the panels. The light colour (light green and light purple) and the “nse level 2” show the results of the level 2 validation. Because the trend was fitted based on logarithmic values, the transformation back to linear values results in a slightly curved shape.


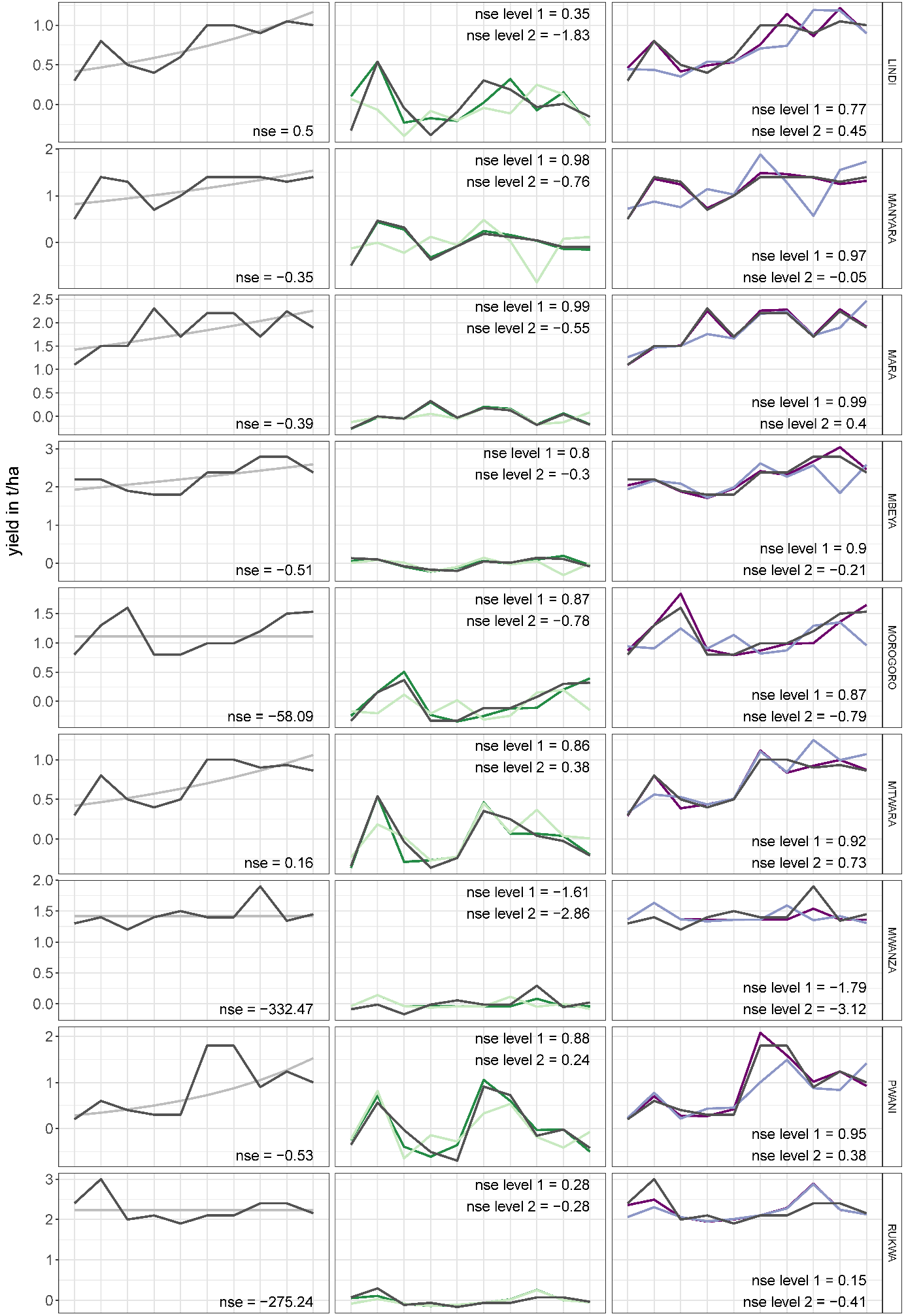


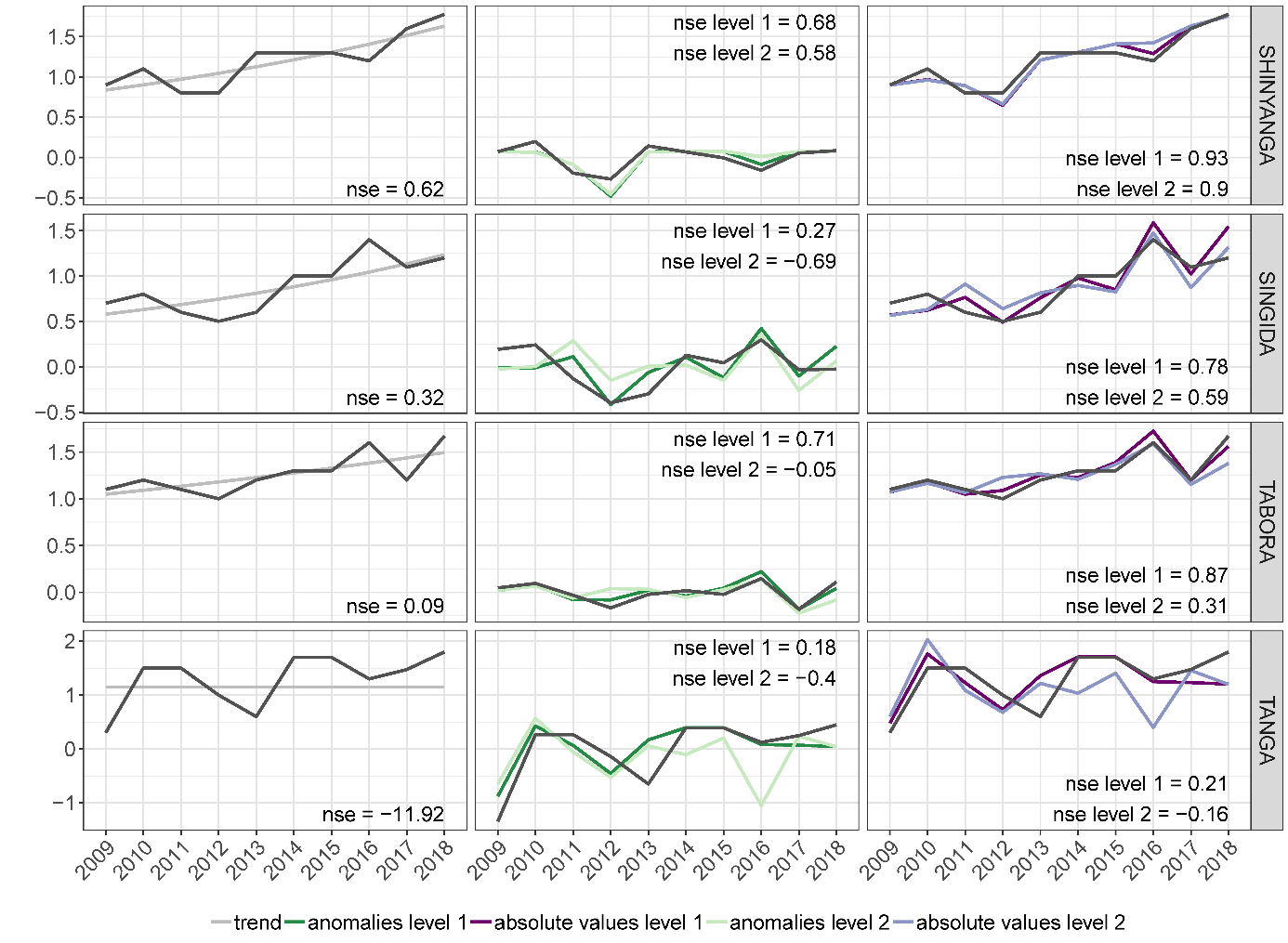


Continuation of Fig. 5


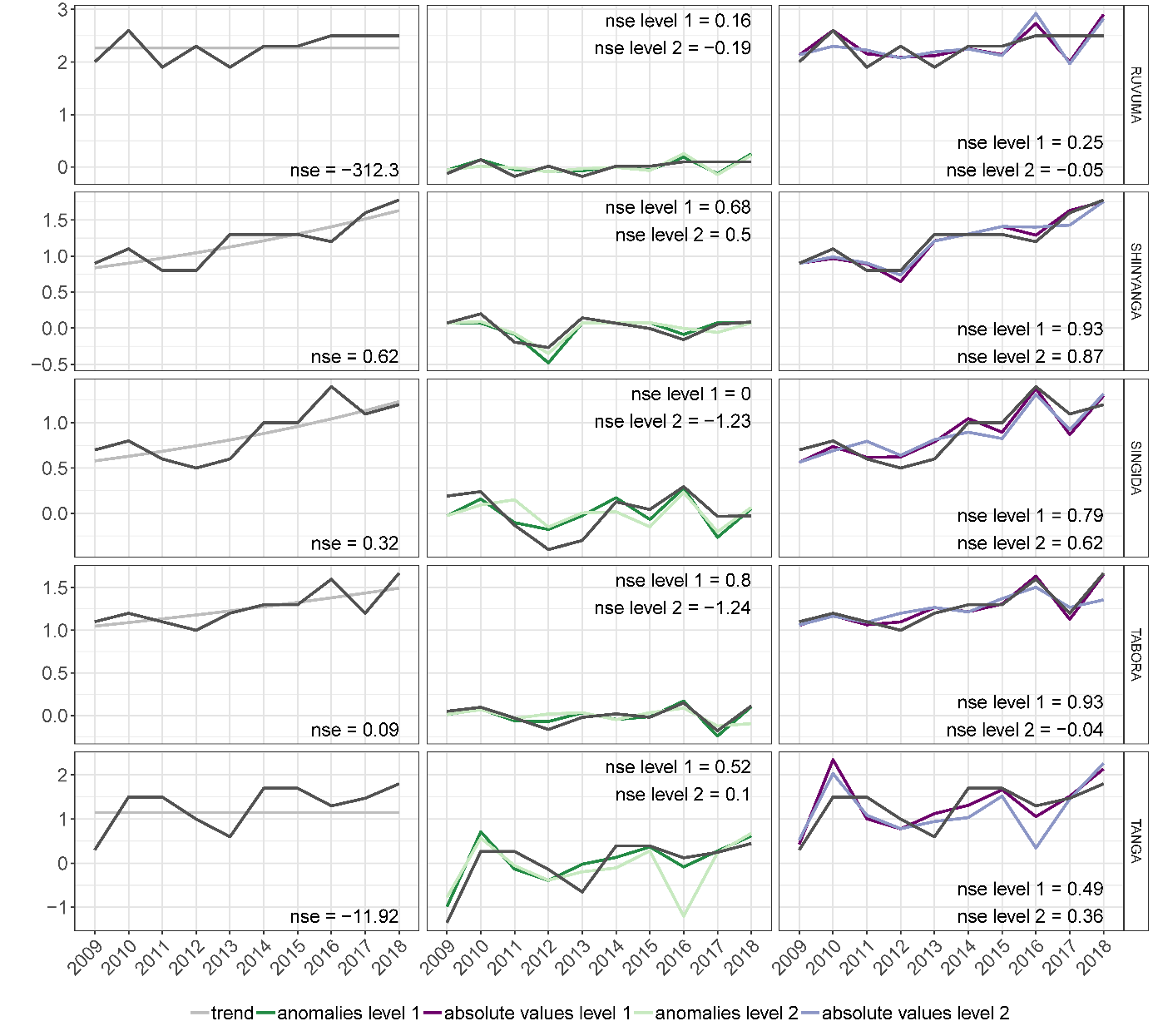


Continuation of Fig. 5

# Strong spatiotemporal variability of maize yields in Tanzania

Maize yields in Tanzania show a high variability and are on a low average level: from 2016 to 2018 maize yields were at 1.6 t/ha on average. The regions with the highest yields (Mbeya, Ruvuma, Rukwa) show the lowest inter-annual variation. The main maize producing regions are Mbeya, Ruvuma, Iringa, Rukwa and Shinyanga).

**
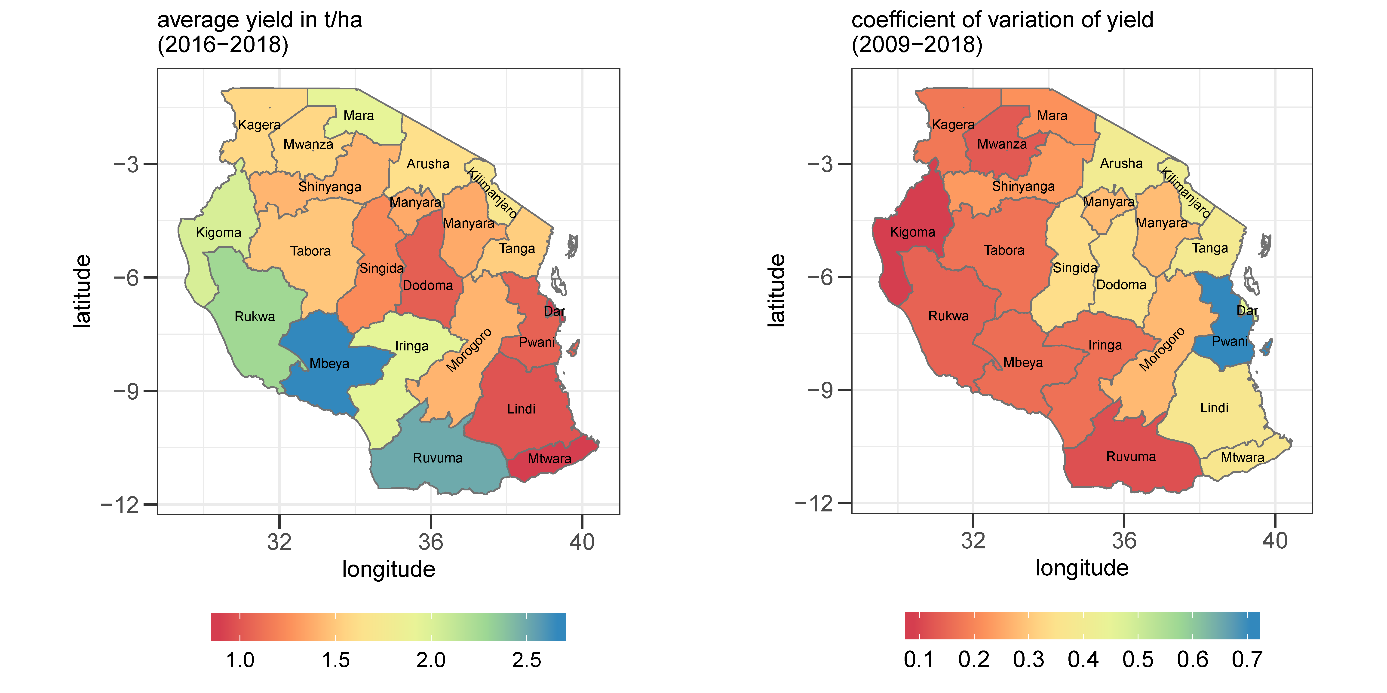
**


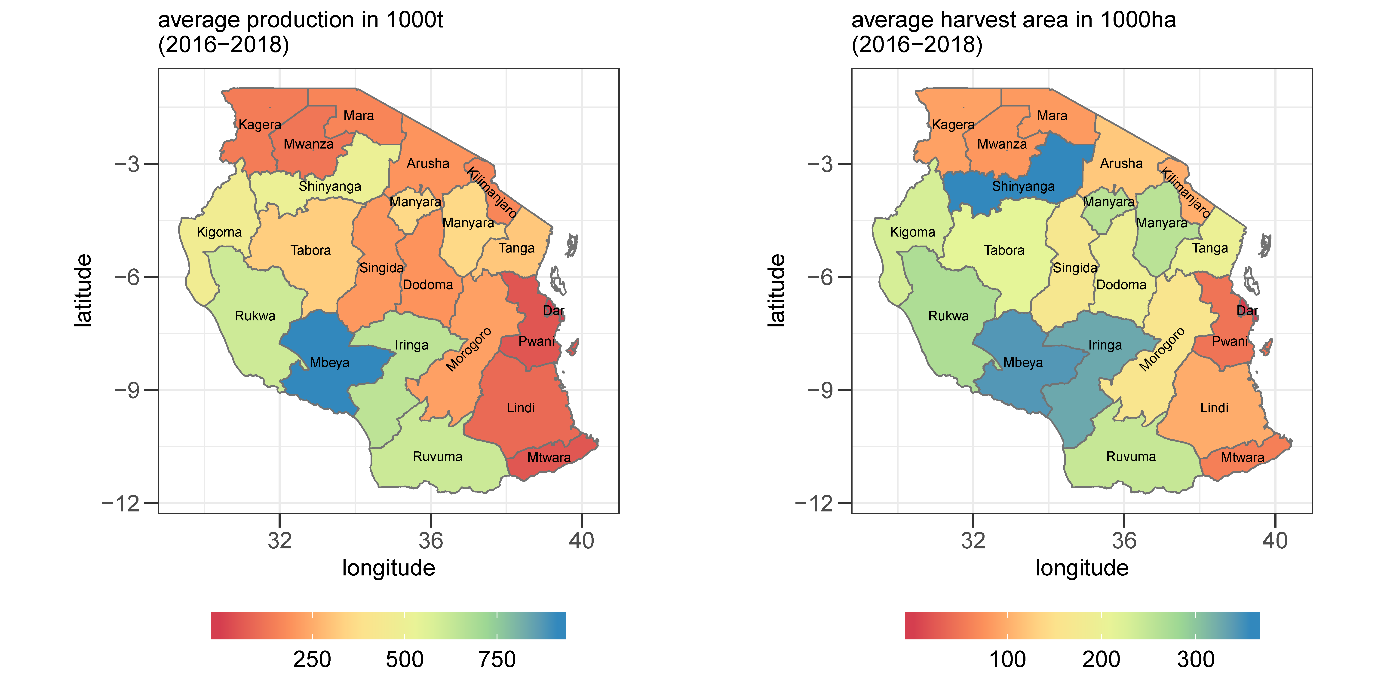


**SI Fig. 6.** Average maize yields, production and harvest area from 2016 – 2018 and the coefficient of variation of yields from all available years (2009-2018).

# Correlation between yield and SST indices with different lead times

We assessed the influence of the following SST (sea surface temperature) indices on Tanzanian yield variability:

1. El Niño 3.4 zone (170°W – 120° W, 5°S - 5°N)
2. West Pacific Box (WP) (130°E – 160°E, 10°S – 10°N)
3. Indian Ocean Dipole (IOD), which is the non-normalised difference between the West Indian Ocean (50°E – 70°E, 10°S – 10°N) and the Eastern Indian Ocean (90°E – 110°E, 10°S - 0°N)

To account for the lag in influence of the SST on rainfall in East Africa, we tested different lag times (0, 30, 60, 90, 120, 150 and 180 days). The strongest influence of SST in the El Niño 3.4 zone on yields in Tanzania can be found at a lead time of 90 and 120 days (SI Fig. 4). There is a positive correlation between yields and the median SST in El Niño 3.4, which could be explained by higher rainfall amounts during the short rains in East Africa in relation with a higher El Niño 3.4 SST ^1–3^. Extreme high and low values of the SST in El Niño 3.4 (values above the 99% and below the 1% percentiles) are related with lower maize yields in Tanzania.

Like the SST in El Niño 3.4, the IOD is also positively correlated with rainfall in East Africa during the short rains ^1–3^. For most regions, the IOD shows the strongest correlations at a lead time of 30 days. At this lead time, the median IOD shows a negative correlation with Tanzanian yield variability. Values below the 1% percentile are positively correlated with yields. At other lead times, the correlation patterns show different directions and the IOD, in contrast to El Niño 3.4 and WP, has the highest region-to-region variability.

The median SST in WP is negatively correlated with maize yield variability in Tanzania. Whereas high values of the SST in WP do not show a clear direction of the correlation, yields tend to be higher in Tanzania the more often the SST falls below the 1%percentile. This can be related to the negative correlation of SST in WP with East African rainfall during the long rains ^1–3^. The highest correlation can be found at a lead time of 90 and 120 days.


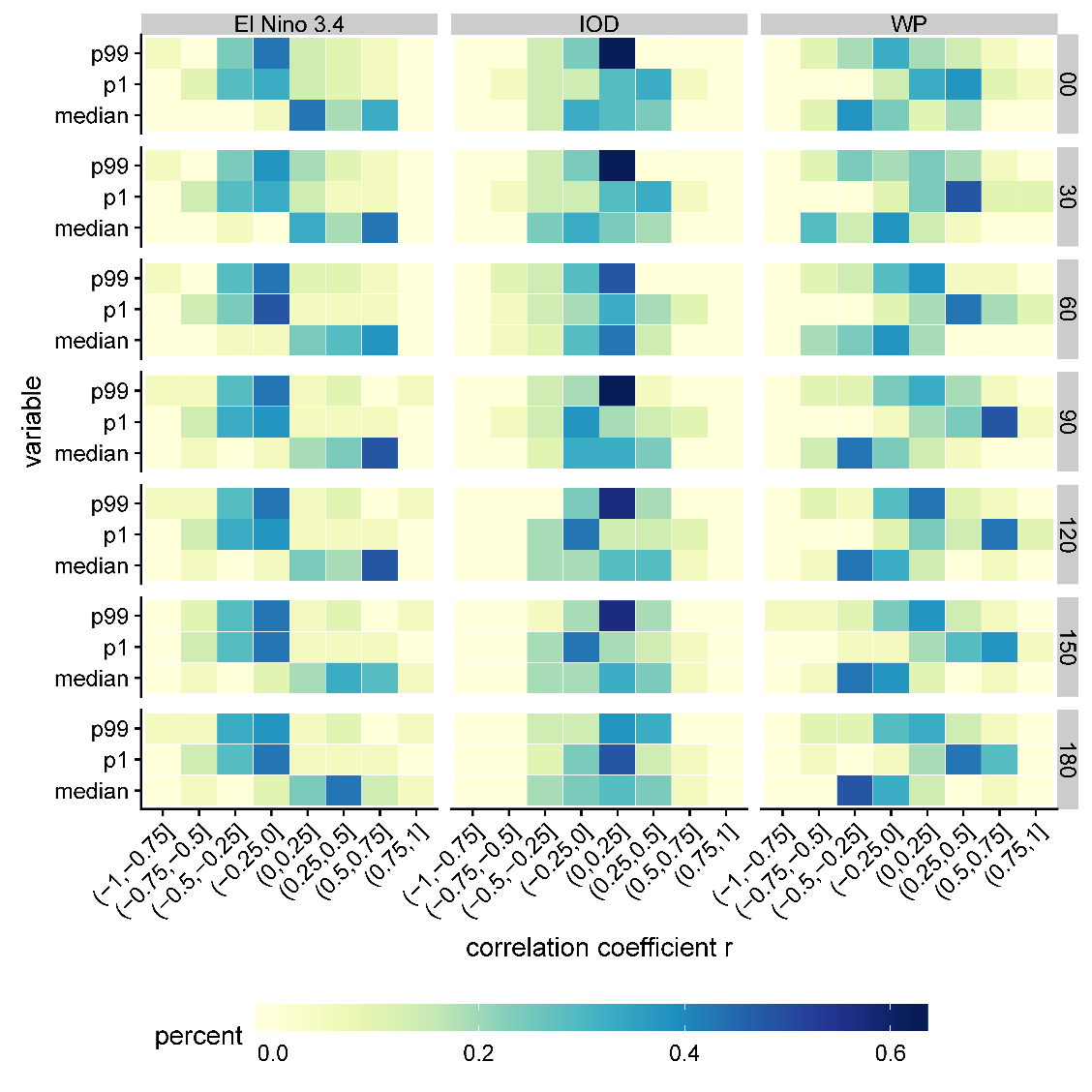


**SI Fig. 7.** The distribution plot shows the correlation coefficients (Pearson’s r) between the demeaned and detrended yields and different standardised SST indices (median, number of times the SST is above the 99% percentile and below the 1% percentile). For a better overview, the correlation coefficients were categorized in classes of 0.25 ranges, so from -1 to -0.75, from -0.75 to -0.5 and so on. The colour indicates how many regions of 21 regions in Tanzania have a particular correlation range between yield and the SST index, e.g. dark blue indicates that 60% of regions (i.e. ca. 13 regions of 21 regions) show a correlation between yield and the SST index of e.g. between 0 and 0.25. The correlation matrix is presented separately for each SST index (horizontally) and the lead times 0, 30, 60, 90, 120, 150 and 180 days (vertically).

# Maize growing season in Tanzania

To define the start of the growing season, we tested the several crop calendars and decided to use the approach of Dodd and Jolliffe (2001) ^4^ that proved to be most suitable for the purpose of our study. In the following paragraphs, we discuss advantages and disadvantages of different crop calendars for maize production in Tanzania.

## FAO crop calendar

The FAO crop calendar from 2012 provides crop-specific planting and harvesting months and the growing season length on district level ^5^. The calendar does not distinguish between the short and the long rains in the bimodal rainfall areas in the North and in the Coastal regions in Tanzania, even though both rainfall seasons are used for crop production ^6^. Moreover, the calendar seems to have unreasonable outliers: Most parts of the Ruvuma region have a growing season from May to August, which is within the dry season. Also the district Sumbawanga Urban in Rukwa has a growing season length of 180days, in contrast to the other parts of the country that have a growing season length of 90 or 120 days.


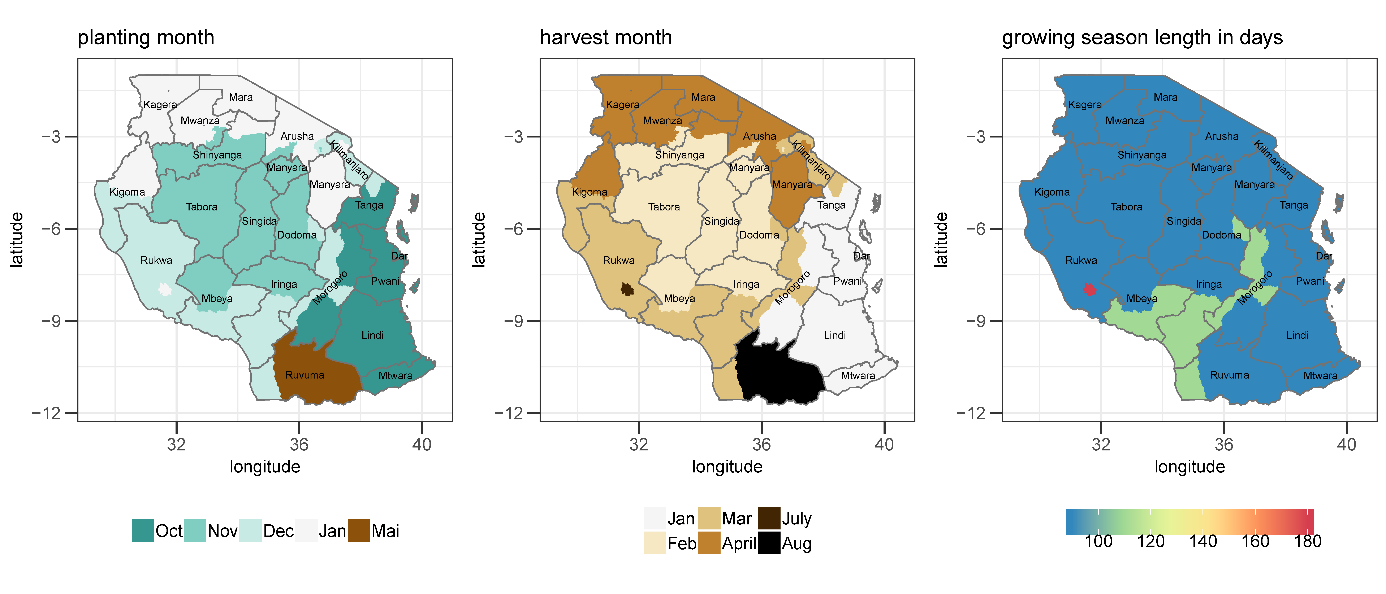
**SI Fig. 8.** Crop calendar for maize based on the FAO crop calendar from 2012 ^5^, planting is defined as the onset of planting and harvest is defined as the onset of harvest.

## FEWS Net crop calendar

The Famine Early Warning Systems Network (FEWS-NET) provides a crop calendar for Tanzania ^7^ that distinguishes between the unimodal and bimodal rainfall regions and the calendar seems to be aligned with the onset of the rains. However, the calendar is neither crop nor region-specific and therefore does not account for the spatial heterogeneity within Tanzania.

## Crop calendar based on Stern et al. (1981)

We calculated the onset of the growing season based on the approach of Stern et al. (1981) ^8^ that was developed for Ghana and Burkina Faso. They define the onset of the rainy season when the following three criteria are met:

1. at least 25 mm rainfall within 5 days
2. starting day and at least two other days in this 5-day period are wet (>0.1 mm)
3. no dry period of seven or more consecutive days within the next 30 days

We defined the end of the growing season as 110 days after the start. Mourice et al., (2014) determined crop specific parameters for maize cultivars in Tanzania based on field experiments and concluded that the cultivars did not differ significantly in terms of the growing season length (they ranged from 105 to 114). Therefore, we use the average growing season length over the considered cultivars, which is about 110 days.

Because of the bimodal rainfall pattern, some regions have two growing seasons. We consider the second growing season if the time between the start of the first growing season and the start of the second growing season is at least 110 days, enough time for one growth cycle.

The criteria of Stern et al. (1981) ^8^ were developed for Ghana and Burkina Faso. For Tanzania, the criteria seem to be too strict – leading to too late onsets of the growing season in particular in the unimodal rainfall areas. Also, in some regions no onset could be calculated for some years, e.g. in Dar es Salaam in 2008 and 2009 and in Manyara in 2009.


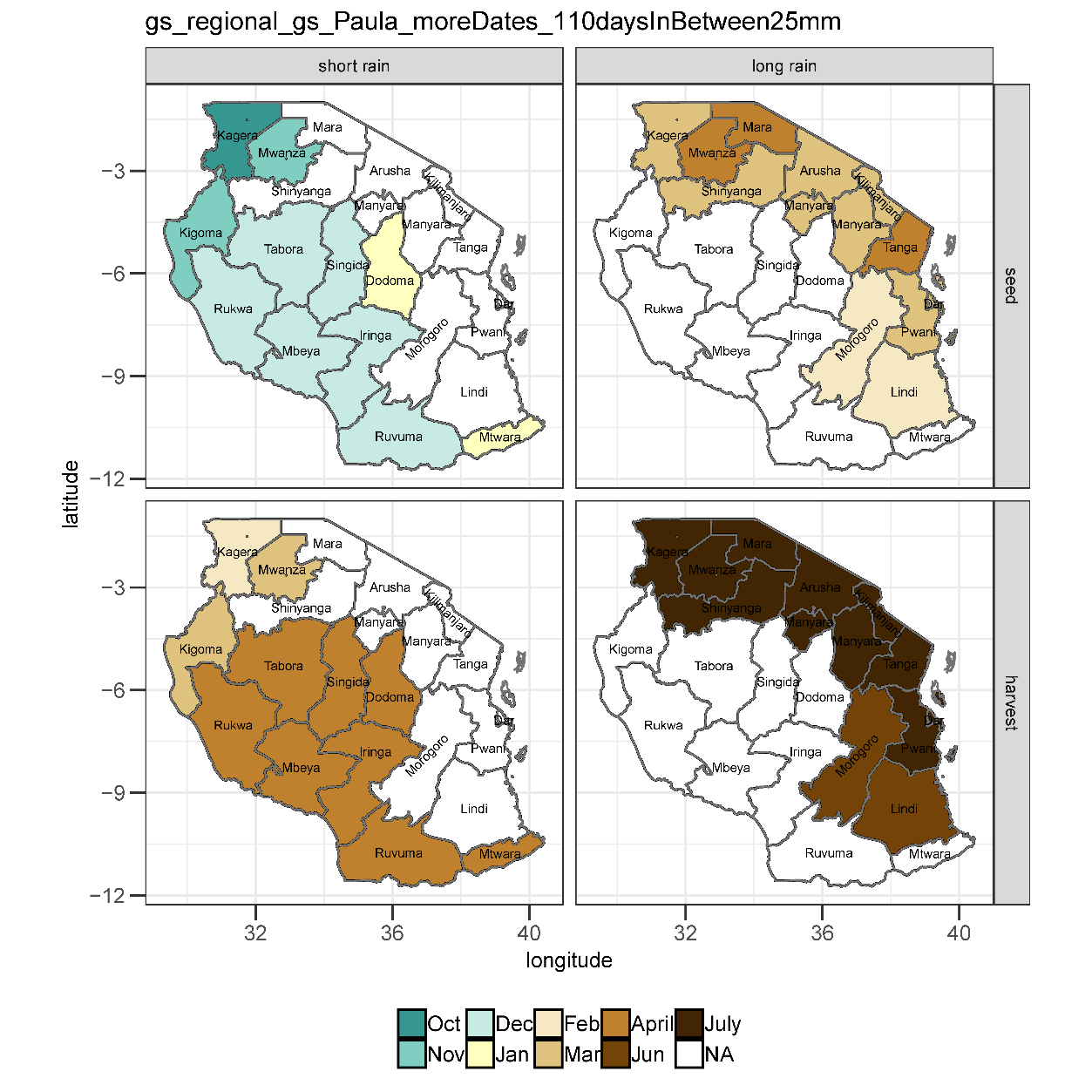


**SI Fig. 9.** Crop calendar for the short (left) and long rains (right) in Tanzania; the onset of the growing season (top row) is calculated based on the approach of Stern et al. (1981). The harvest dates (bottom row) represent the sowing dates plus the maize specific growing season length of 110 days according to Mourice et al., (2014). The figure shows the median sowing and harvesting dates over the period from 2009 to 2018 for each region in Tanzania.

## Crop calendar based on Dodd and Jolliffe (2001)

Dodd and Jolliffe (2001) ^4^ further developed the approach of Stern et al. (1981) ^8^ to correct for too late onsets of the growing season. Their approach was tested for tropical and subtropical conditions. According to Dodd and Jolliffe (2001) ^4^, the onset of the growing season is defined when the following three criteria are fulfilled:

1. at least 25 mm rainfall within 6 days

2. starting day and at least two other days in this 6-day period are wet (>0.1 mm)

3. no dry period of ten or more consecutive days within next 40 days

Because of the bimodal rainfall pattern in North and North-East Tanzania, two onsets of the growing season are found for some grid points. In this case, we considered the onset of the long rains (Masika), which is the main growing season.

Following Mourice et al., (2014) ^9^, we consider a growing season length of 110 days, as described in section 7.3.


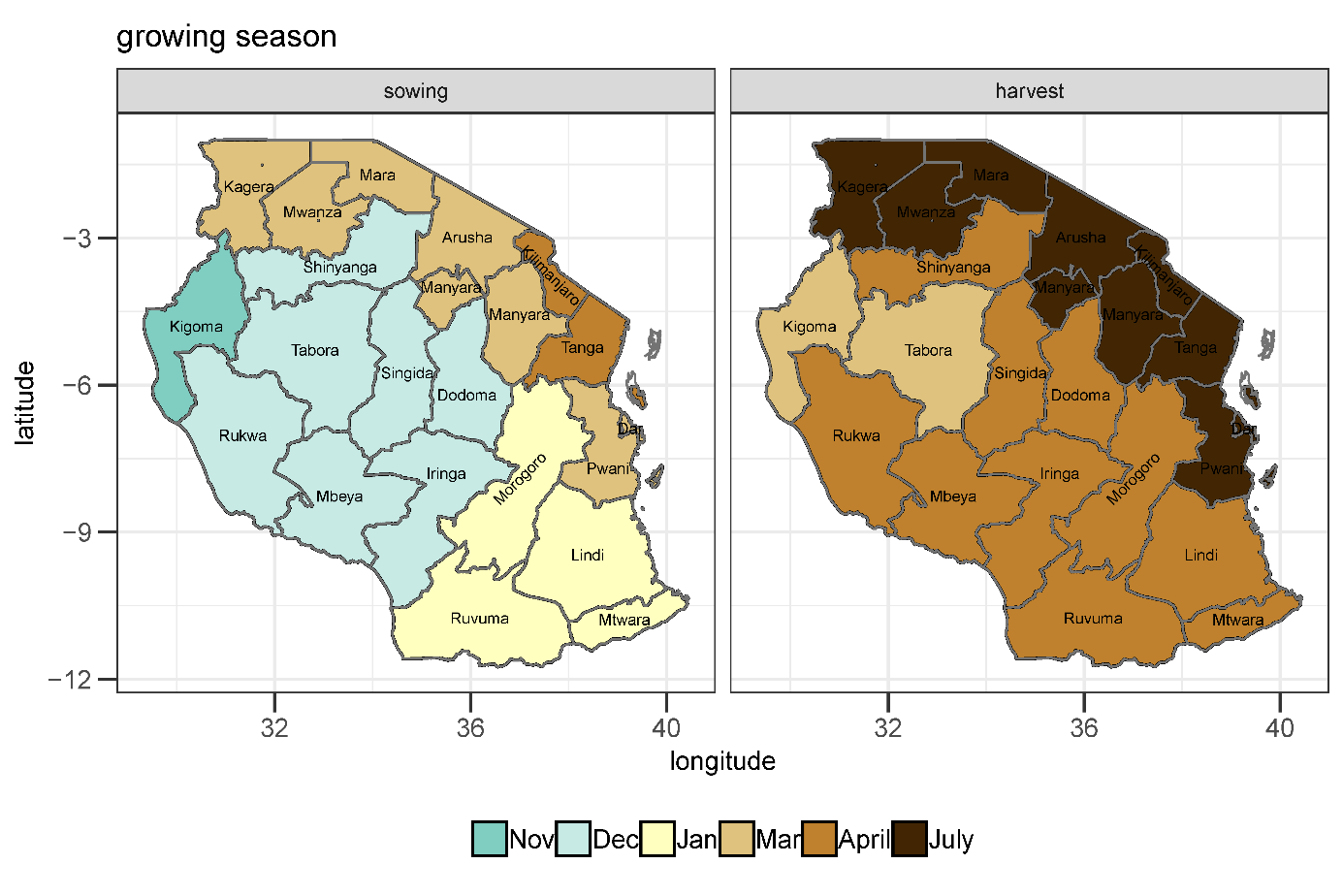


**SI Fig. 10.** Sowing (left) and harvest (right) dates for Tanzania; the onset of the growing season is calculated based on the approach of Dodd and Jolliffe (2001) ^4^. The harvest dates represent the sowing dates plus the maize specific growing season length of 110 days according to Mourice et al., (2014). In case of two calculated rainy seasons, we considered the onset of the long rains (Masika), which is the main growing season. The figure shows the median sowing and harvesting dates over the period from 2009 to 2018 for each region in Tanzania.

# Equations for the calculation of GDD and percentile variables

**SI Eq. 1. C**alculation of percentile variables; *d* denotes the number of days within the growing season; *Days* denotes the total number of days within growing season

$var.p99=\sum_{d=1}^{Days} {var}_{d}^{var.p99}$ ${var}_{d}^{var.p99}=\left\{ \begin{aligned} 1, &{var}_{d}> p.99 \\ 0, &otherwise \end{aligned} \right.$

$var.p01=\sum_{d=1}^{Days} {var}_{d}^{var.p01}$ ${var}_{d}^{var.01}=\left\{ \begin{aligned} 1, &{var}_{d}< p.01 \\ 0, &otherwise \end{aligned} \right.$

With $var$ as the weather or sea surface temperature (SST) variable, and $p.99$ ($p.01$) as the 99% (1%) percentile of the weather or SST variable; the percentiles were calculated over all days of the vegetative and reproductive phase of the growing season within the time period of 2009 and 2018 for each region. As a sensitivity test, we also calculated the 5% (95%) and 10% (90%) percentiles, which provided similar results.

**SI Eq. 2.** Calculation of growing degree days (GDD); *d* denotes the number of days within the growing season; *Days* denotes the total number of days within growing season

$GDD=\sum_{d=1}^{Days} T_{d}^{GDD}T_{d}^{GDD}=\left\{ \begin{aligned} 0, &T_{d}<T^{Base} \\ T_{d}-T^{Base}, & x\geq T^{Base}\leq T_{d}\leq T^{Opt} \\ T^{Opt}-T^{Base}, & x\geq T_{d}>T^{Opt} \end{aligned} \right.$

With *T* as daily mean temperature; *T^Base^* as base temperature of 10°C; *T^Opt^* as optimal temperature of 30°C ^10^; *d* denotes the number of days within the growing season; *Days* denotes the total number of days within growing season

# Lead time of the yield forecast per region

| region | start vegetative phase | start reproductive phase (days after sowing) | harvest | lead time of forecast in days | Performance of forecast (NSE of the level 1 - LOOCV) |
| --- | --- | --- | --- | --- | --- |
| Dodoma | 29-Dec | 22-Feb (55) | 18-Apr | 55 | 0.9 |
| Arusha | 30-Mar | 19-May (50) | 18-Jul | 60 | 0.97 |
| Kilimanjaro | 03-Apr | 23-May (50) | 22-Jul | 60 | 0.93 |
| Tanga | 01-Apr | 22-May (51) | 20-Jul | 59 | 0.28 |
| Morogoro | 03-Jan | 25-Feb (53) | 23-Apr | 57 | 0.79 |
| Pwani | 28-Mar | 20-May (53) | 16-Jul | 57 | 0.63 |
| Dar es Salaam | 25-Mar | 17-May (53) | 13-Jul | 57 | 0.92 |
| Lindi | 02-Jan | 25-Feb (54) | 22-Apr | 56 | 0.59 |
| Mtwara | 10-Jan | 05-Mar (54) | 30-Apr | 56 | 0.78 |
| Ruvuma | 02-Jan | 25-Feb (54) | 22-Apr | 56 | 0.07 |
| Iringa | 29-Dec | 21-Feb (54) | 18-Apr | 56 | 0.58 |
| Mbeya | 21-Dec | 15-Feb (56) | 10-Apr | 54 | 0.98 |
| Singida | 26-Dec | 20-Feb (56) | 15-Apr | 54 | 0.73 |
| Tabora | 07-Dec | 02-Feb (57) | 27-Mar | 53 | 0.88 |
| Rukwa | 23-Dec | 17-Feb (56) | 12-Apr | 54 | 0.82 |
| Kigoma | 20-Nov | 16-Jan (57) | 10-Mar | 53 | -0.39 |
| Shinyanga | 12-Dec | 07-Feb (57) | 01-Apr | 53 | 0.99 |
| Kagera | 28-Mar | 23-May (56) | 16-Jul | 54 | 0.25 |
| Mwanza | 26-Mar | 20-May (55) | 14-Jul | 55 | 0.19 |
| Mara | 27-Mar | 21-May (55) | 15-Jul | 55 | 0.99 |
| Manyara | 27-Mar | 16-May (50) | 15-Jul | 60 | 0.97 |

**SI Table 5.** Lead time in days of the yield forecast per region; the lead time of the forecast corresponds to the length of the reproductive phase; the start of the vegetative phase (2^nd^ columns) corresponds to 0 days after sowing; the harvest date (4^th^ columns) corresponds to 110 days after sowing; the right column shows the performance of the forecast of absolute yields measured in the NSE of the level 1 validation; unimodal rainfall regions are shaded in blue, bimodal rainfall regions are shaded in yellow

# References

1. Hoell, A. & Funk, C. Indo-Pacific sea surface temperature influences on failed consecutive rainy seasons over eastern Africa. *Clim. Dyn.* **43**, 1645–1660 (2014).

2. Funk, C. *et al.* Predicting East African spring droughts using Pacific and Indian Ocean sea surface temperature indices. *Hydrol. Earth Syst. Sci.* **18**, 4965–4978 (2014).

3. Davenport, F., Funk, C. & Galu, G. How will East African maize yields respond to climate change and can agricultural development mitigate this response? *Clim. Change* **147**, 491–506 (2018).

4. Dodd, D. E. S. & Jolliffe, I. T. Early detection of the start of the wet season in semiarid tropical climates of Western Africa. *Int. J. Climatol.* **21**, 1251–1262 (2001).

5. FAO. FAOSTAT - Food and agriculutre data. (2019). Available at: http://www.fao.org/faostat/. (Accessed: 10th April 2019)

6. Suleiman, R. & Rosentrater, K. Current maize production, postharvest losses and the risk of Mycotoxins contamination in Tanzania. *Am. Soc. Agric. Biol. Eng. Annu. Int. Meet. 2015* **4**, 3289–3414 (2015).

7. FEWS NET. East Africa - Tanzania. (2019). Available at: https://fews.net/east-africa/tanzania. (Accessed: 24th December 2019)

8. Stern, R. D., Dennett, M. D. & Garbutt, D. J. The Start of the Rains in West Africa. *J. Climatol.* **1**, 59–68 (1981).

9. Mourice, S. K., Rweyemamu, C. L., Tumbo, S. D. & Amuri, N. Maize Cultivar Specific Parameters for Decision Support System for Agrotechnology Transfer (DSSAT) Application in Tanzania. *Am. J. Plant Sci.* **05**, 821–833 (2014).

10. Gilmore, E. C. & Rogers, J. S. Heat Units as a Method of Measuring Maturity in Corn. *Agron. J.* **50**, 611–315 (1958).
